# Three new species of *Heteromysis* (Mysida, Mysidae, Heteromysini) from the Cape Peninsula, South Africa, with first documentation of a mysid-cephalopod association

**DOI:** 10.3897/zookeys.685.13890

**Published:** 2017-07-13

**Authors:** Karl J. Wittmann, Charles L. Griffiths

**Affiliations:** 1 Abteilung für Umwelthygiene, Zentrum für Public Health, Medizinische Universität Wien, Kinderspitalgasse 10, A-1090 Vienna, Austria; 2 Department of Biological Sciences and Marine Research Institute, University of Cape Town, Rondebosch 7701, Republic of South Africa

**Keywords:** Crustacea, hermit crab association, octopus association, ectocommensals, taxonomy, key to species, SE. Atlantic

## Abstract

Faunistic studies in sublittoral and littoral marine habitats on the Cape Peninsula, South Africa, have yielded three new species belonging to the genus *Heteromysis*, subgenus Heteromysis: *H. cancelli* **sp. n.** associated with the diogenid hermit crab *Cancellus macrothrix* Stebbing, 1924, and *H. fosteri* **sp. n.** extracted from ‘empty’ urchin and gastropod shells. The first documented mysid-cephalopod association is reported for *H. octopodis* **sp. n.** which was found in dens occupied by *Octopus vulgaris* Cuvier, 1797, but was also captured from tide pools. The three new species differ from previously known E. Atlantic species, among other characters, by a single spine on the endopods of uropods in combination with large cornea and absence of median sternal processes on thoracic somites. They are also characterized by a white stripe along the dorso-lateral terminal margin of the eyestalks in living specimens. The new species appear quite similar to each other, but are distinguished by different depths of the telson cleft, different distributions of spines on the lateral margins of the telson, different numbers of segments on thoracic endopod 4, and by differently modified setae on the carpus of the third thoracic endopod, as well as on the carpopropodus of the fourth endopod. An updated key to the species of *Heteromysis* known from the E. Atlantic is given.

## Introduction

Ongoing taxonomic surveys of littoral and sublittoral habitats along the coasts of the Cape Peninsula (South Africa) have yielded a number of previously unreported Mysidae species, of which one, *Mysidopsis zsilaveczi*, has already been described as new by Wittmann and Griffiths (2014). The examination of this material is now continued with descriptions of three new species of *Heteromysis* S.I. Smith, 1873. Not counting doubtful species and non-nominotypical subspecies, but including the new ones described here, as many as 88 species of this genus are currently acknowledged on a world-wide scale and lots of additional new ones may be expected. Four subgenera are distinguished. However, as many as 28 species have so far not been assigned to any subgenus (Mees 2017), particularly due to missing data. This is also the case for two E. Atlantic species given in the key below. Including the three new ones, all 11 currently known species from the E. Atlantic are from cold-temperate to subtropical climates. Elsewhere, the main bulk of species comes from subtropical to tropical climates. In this view, the coastal waters of the tropical E. Atlantic appear as practically unexplored for this genus. This is true also for the temperate SE. Atlantic, as no *Heteromysis* species have been previously described from South Africa, although for the sake of completeness we note that Connell (1974) reported a single specimen of *Heteromysis* sp., without giving any further details, from the Mtentu River Estuary on the Indian Ocean coast of South Africa.

The distributions and habitats occupied by species belonging to this genus were reviewed by Fukuoka (2005). Most species were found in cryptic habitats, such as dense vegetation, microcaves, empty gastropod shells, etc. Many species are also associated with benthic invertebrates, some are known even as obligate commensals, at least during daytime. The present paper adds one species associated with hermit crabs, an additional one from empty shells, and a third one associated with octopus, with this last association documented as the first known facultative ectocommensal symbiosis between mysids and cephalopods.

## Material and methods

The three species described below were collected at four stations in 0-20 m depth within 5.5 km of the False Bay coastline (34°11'S 18°27'E to 34°14'S 18°28'E), Cape Peninsula, South Africa, using different methods, as detailed below:

The first species was extracted from a gastropod shell occupied by a hermit crab, as part of a separate taxonomic study on the regional hermit crab fauna. Although numerous shells, occupied by a variety of hermit crab species, were collected individually by SCUBA diving or by hand-collecting on the shore at this and many other sites, mysids were only found associated with a single individual of the rarely-recorded hermit *Cancellus macrothrix* Stebbing, 1924.

The second, shell-occupying species was discovered during a separate and mysid-directed sampling operation, in which roughly 1 kilo of dead urchin and gastropod shells, without hermit crabs, were sampled from sublittoral habitats near Miller’s Point, False Bay, then put into a bucket of seawater, into which a small amount of formalin was added, just enough to expel any infauna from the recesses, but not to kill them. The sample was then rinsed with seawater and each shell shaken out back into the bucket. The seawater was then poured through a 1 mm mesh net. Only the single mysid species described below was obtained in this way (although along with numerous amphipods, isopods and other small crustaceans).

The third species described here was visually sighted and photographed in sublittoral dens occupied by octopus and in intertidal rock pools by a local underwater photographer, who then captured specimens with a small hand net, or by coaxing them into an open jar, and submitted them to us for identification.

Fixation, preservation, preparation, measurements, and examination of materials was carried out as described in Wittmann (2000); and examination of statolith structure as detailed in Wittmann et al. (1993). Terminology of larval stages is given according to Wittmann (1981). With certain modifications, as stated by Wittmann (2000), appendage terminology is according to Tattersall and Tattersall (1951), and for non-sensory cuticle structures according to Klepal and Kastner (1980). Holotypes and slides of dissected paratypes were deposited at the Natural History Museum of Vienna (NHMW), vial paratypes at the Iziko South African Museum, Cape Town (SAM).

## Systematics

### Order Mysida Boas, 1883

#### Family Mysidae Haworth, 1825

#### Subfamily Heteromysinae Norman, 1892

#### Tribe Heteromysini Norman, 1892

#### Genus *Heteromysis* S.I. Smith, 1873

#### Subgenus Heteromysis S.I. Smith, 1873

#### Heteromysis (Heteromysis) cancelli

Taxon classificationAnimaliaMysidaMysidae

Wittmann & Griffiths sp. n.

http://zoobank.org/A2B8C8A8-CC29-4464-8E01-841454C06837

**Figure 1.**
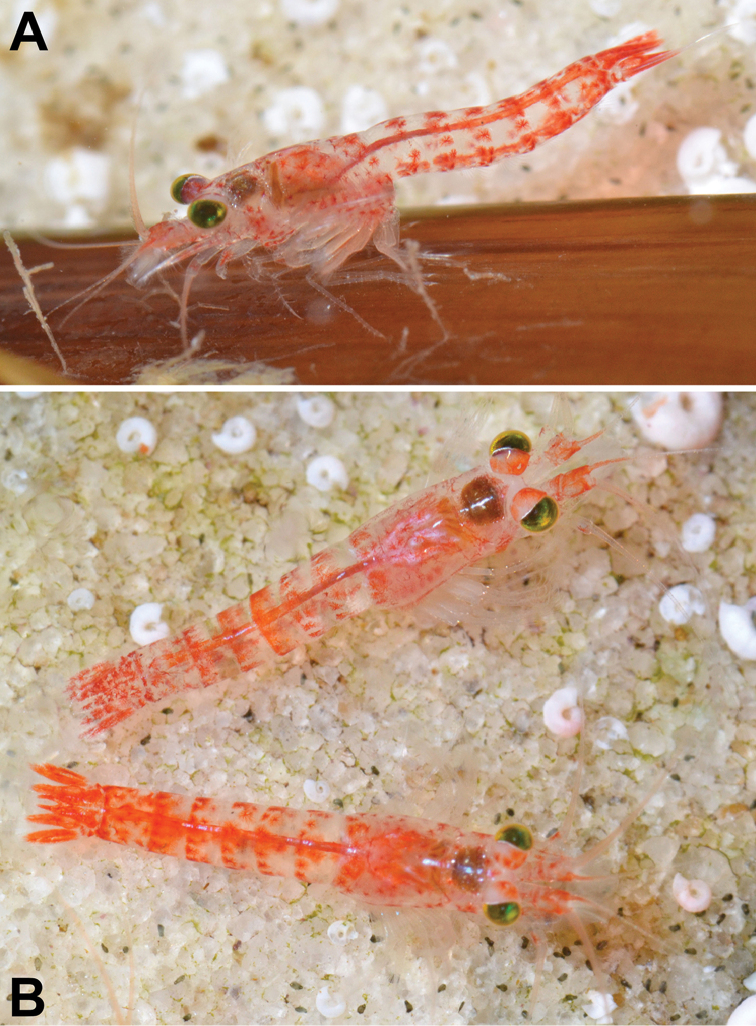
Ex-situ microphotographs of *Heteromysis cancelli* sp. n. from False Bay, Simonstown, Cape Peninsula, South Africa; adult males with 6–7 mm body length in lateral (**A**) or in dorsal view (**B**), respectively. Aquarium photos by C.L. Griffiths.

**Figure 2.**
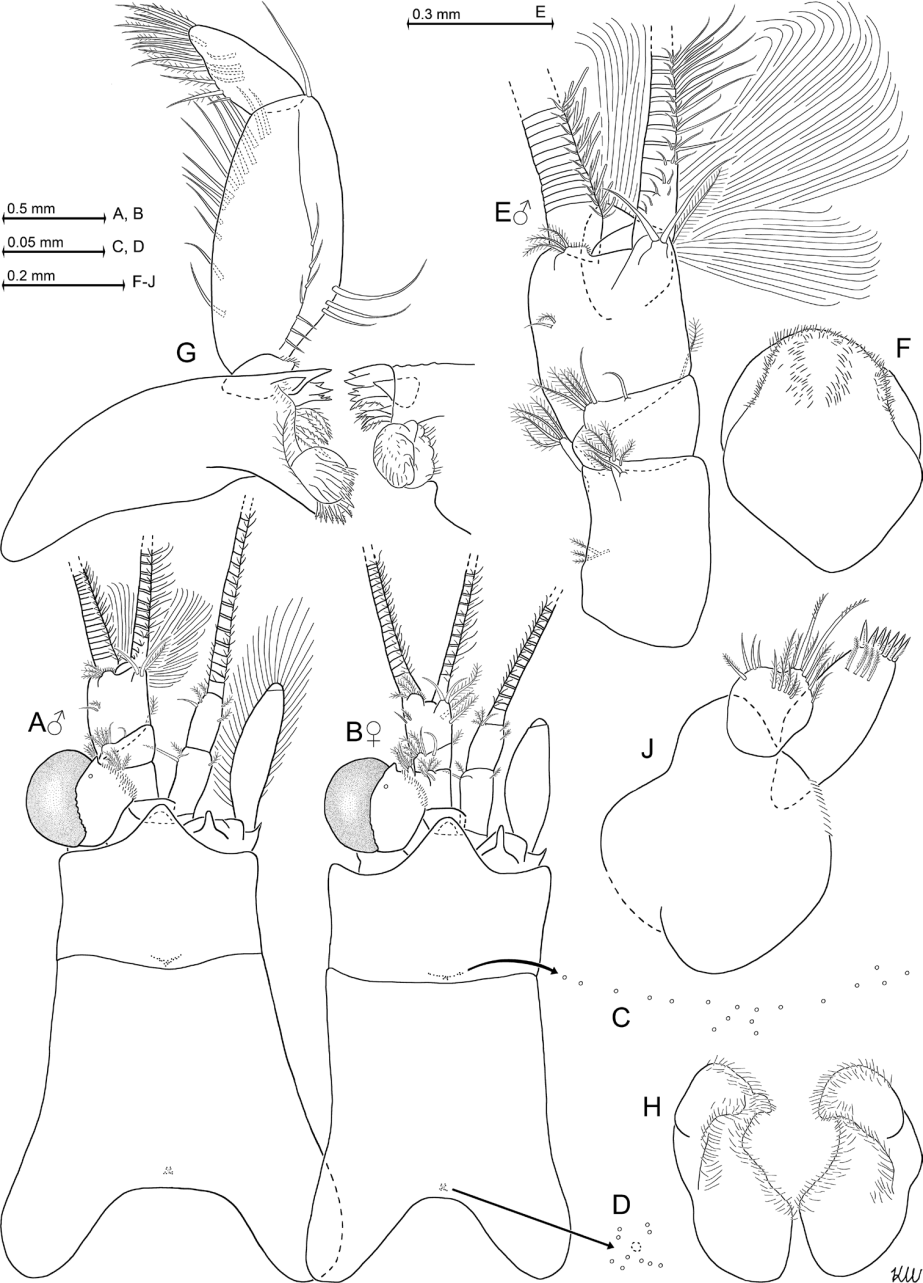
*Heteromysis cancelli* sp. n., paratype male with 6.9 mm body length (**A, E–H**), paratype female 6.4 mm (**B–D, J**). **A, B** cephalic region plus carapace in male (**A**) versus female (**B**), dorsal view, details show pore groups (**C, D**) on carapace **E** male antennula, dorsal **F** labrum, obliquely ventral **G** mandibles with right palpus, caudal **H** labium, ventral **J** maxillula, caudal.

**Figure 3.**
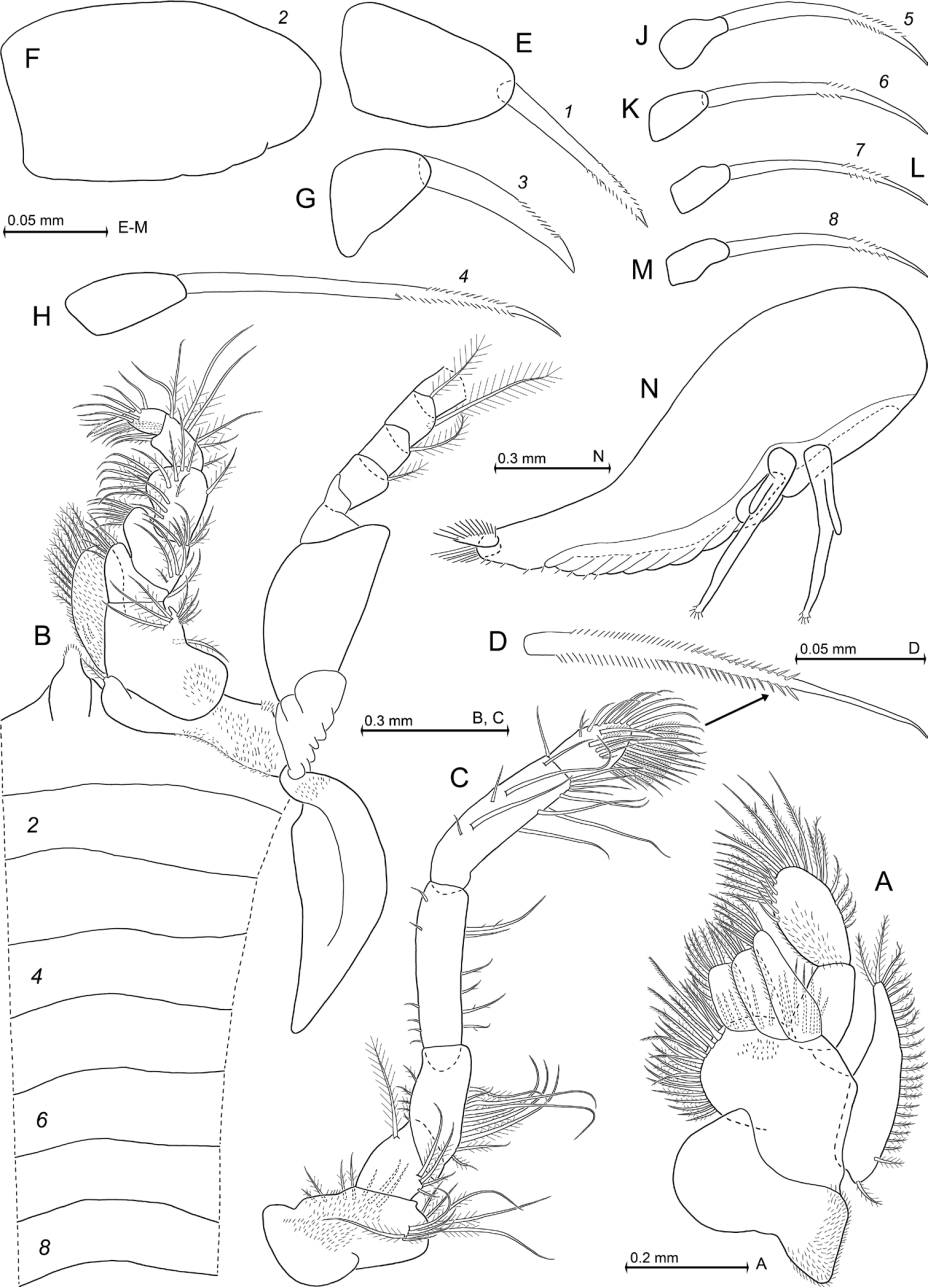
*Heteromysis cancelli* sp. n., paratype female with 6.4 mm body length (**A, C, D, H, J, L, M**) with one of its larvae (**N**), paratype male 6.9 mm (**B, E–G, K**). **A** maxilla, caudal **B** first thoracopod, caudal aspect, with thoracic sternites 1-8, ventral **C** second maxilliped, ventral, detail (**D**) shows modified seta **E–M** series of dactylus in thoracic endopods 1-8 with claw (if present), caudal **N** nauplioid larva at late substage 2, lateral.

**Figure 4.**
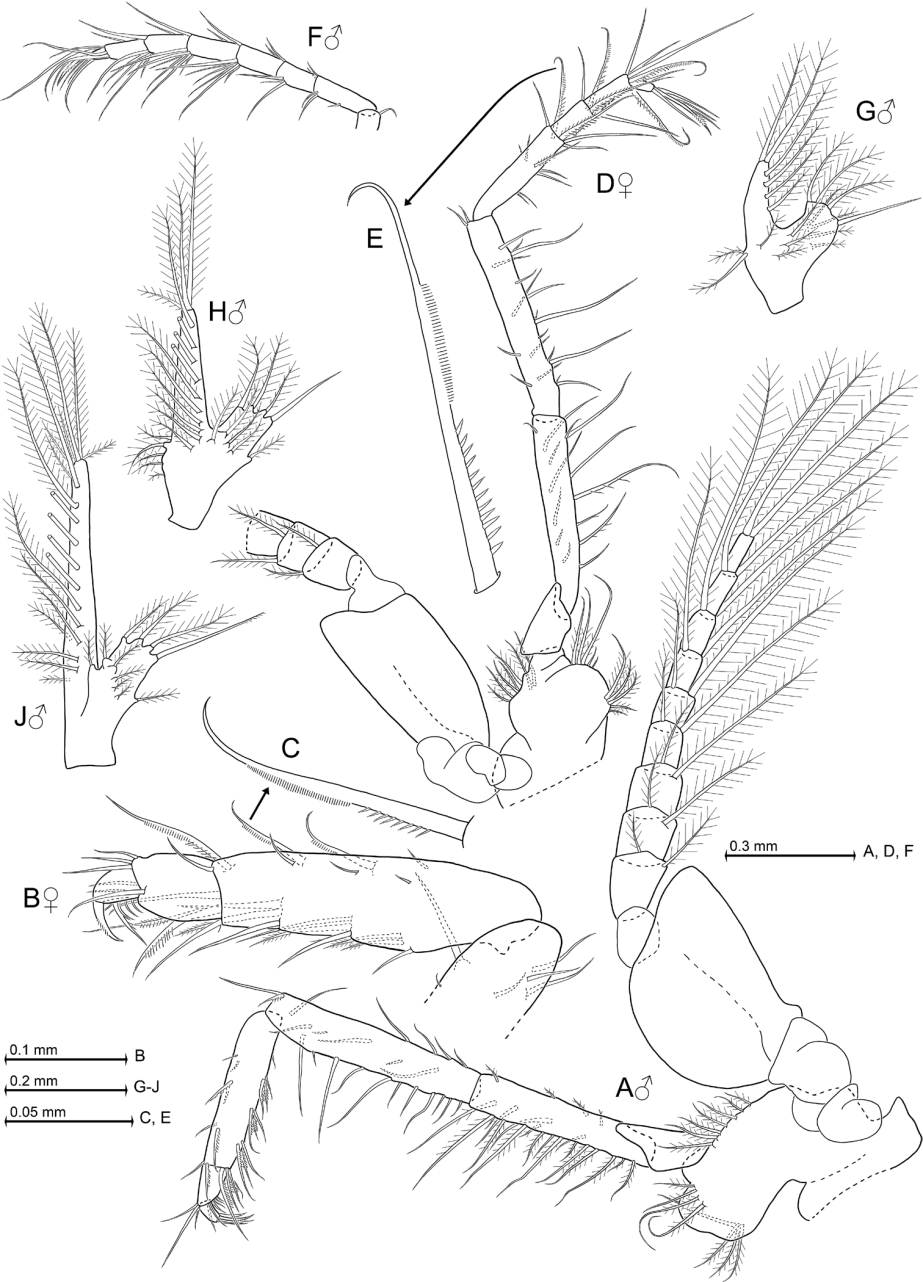
*Heteromysis cancelli* sp. n., paratype male with 6.9 mm body length (**A, F–J**), paratypes female 6.1 mm (**B, C**) and 6.4 mm (**D, E**). **A** male third thoracopod, caudal aspect **B** tarsus of female thoracic endopod 3, rostral, detail (**C**) shows modified seta **D** fourth thoracopod, rostral, detail (**E**) shows modified seta **F** tarsus of sixth thoracic endopod, rostral **G–J** series of male pleopods 1, 4, 5, rostral.

**Figure 5.**
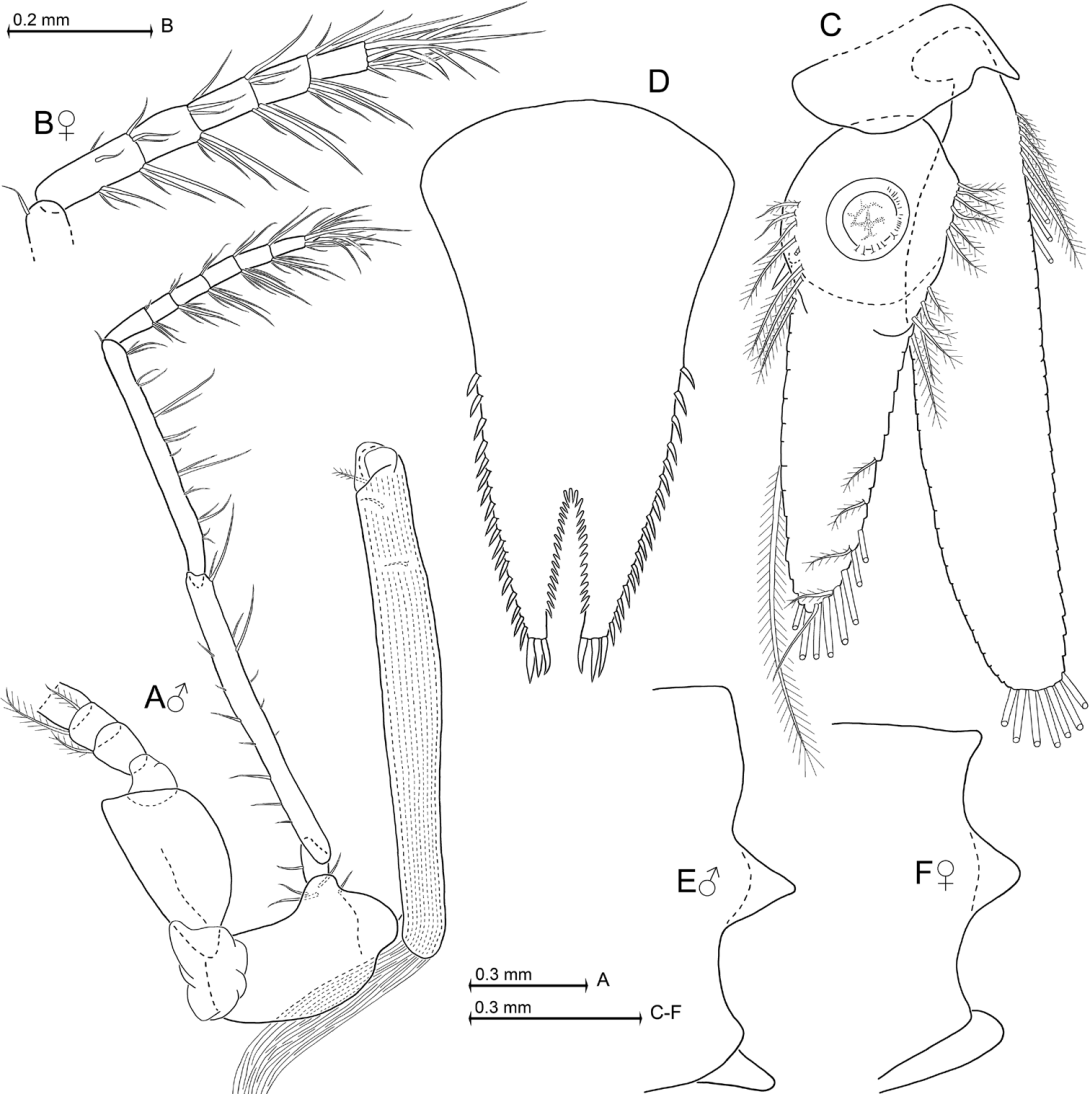
*Heteromysis cancelli* sp. n., paratype male with 6.9 mm body length (**A, C–E**), paratype female 6.4 mm (**B, F**) **A** eighth thoracopod with penis, caudal aspect **B** tarsus of female eighth thoracic endopod, caudal **C** uropods, dorsal **D** telson, dorsal **E, F** terminal margin of sixth pleonite, lateral, in male (**E**) versus female (**F**).

Figs 1 , 2 , 3 , 4 , 5

##### Type series.

Holotype, adult male with 6.9 mm body length, in vial at NHMW-25903; paratypes in vial at SAM-MB-A067550: adult male 5.3 mm, adult female 6.1 mm, subadult female 6.0 mm, one immature female, one juvenile; dissected paratypes on slides at NHMW-25904: adult female 6.4 mm and adult male 6.9 mm; Roman Rock, off Simonstown, on the False Bay coastline of the Cape Peninsula, South Africa, 34°10.95'S, 18°27.50'E, 20 m depth; all eight specimens from the same gastropod shell inhabited by the hermit crab *Cancellus macrothrix* Stebbing, 1924, 3 May 2015, leg. Jannes Landschoff.

##### Diagnosis.

Carapace produced into well projecting, broadly-subtriangular rostrum with rounded apex. Eyes well developed; cornea occupies 40–55% of eye surface; eyestalks with small, distally directed, blunt extension of (obliquely anterior facing) inner margin. Antennular trunk with a number of smooth and barbed setae, but no particularly modified setae; inner distal corner of its terminal segment with anteriorly directed apophysis carrying two large, smooth setae. Antennal scale moderately stout and short, extending shortly beyond distal half of terminal segment of antennular trunk; outer margin feebly convex. First thoracic sternite with anteriorly projecting, terminally rounded median lobe; sternites 2–8 without lobes in both sexes. Carpopropodus of thoracic endopods 1–8 with 2, 2, 2, 3-4, 5, 6, 6, or 5-6 segments, respectively. Third thoracic endopod not dimorphic, without any spines or spine-like setae; carpus not swollen (with respect to merus). Carpus of third thoracic endopod with series of 2–3 subbasally spiny (i.e. with modified barbs) and medially to subterminally pectinate setae near outer margin. Carpopropodus of fourth thoracic endopod with series of 2–4 subbasally more strongly spiny setae near outer margin, these ‘spines’ thick but not tooth-like; no such modified setae in endopods 5–8. Penes long and slender, twice length of merus of eighth thoracic endopod; tip with three rounded lobes, each wider than long. Pleopods reduced to small, setose, bilobate plates, without any spines in both sexes. Exopods of uropods extend distinctly beyond endopods. Endopods with only one spine on inner margin, in subbasal position near statocyst. Telson subtriangular, though terminally transversely truncate; lateral margins weakly sigmoid, along their distal 56–60% furnished with continuous series of 18–21 spines each. Telson with apical cleft that forms a narrow, proximally rounded ‘V’, cleft four times deeper than wide, its depth 27–36% telson length. Cleft densely furnished with total of 29–33 acute laminae (= laminar processes) along basal 81–84% of its margins. Two latero-apical lobes of telson show transverse apical margins, each carrying 2–3 large spines of subequal size.

##### Description.

General appearance is that of mysids with intermediate proportions. Cephalothorax comprises 39–42% of body length without telson, pleon 58–61%, and carapace 33–37%, when measured along dorsal median line (Fig. 1). First thoracic sternite with median lobe showing a number of minute bristles on its well-rounded apex (Fig. 3B). Each of first to fifth abdominal somites measures 0.7–0.9 times length of sixth somite. Terminal margin of sixth pleonite with sinusoidal lateral shields covering the basis of uropods in females (Fig. 5F) versus (sub)triangular ones in males (Fig. 5E).

***Carapace*** (Fig. 2A, D). Non-dimorphic, antero-lateral edges evenly rounded. Cervical sulcus well marked, no cardial sulcus visible. Posterior margin rounded, emarginated, leaving the ultimate and part of penultimate thoracic segment dorsally exposed. Carapaces dissected and mounted on slides in only a single specimen for each sex: 10–11 pores (Fig. 2D) of about 1 µm diameter are in roughly butterfly-shaped arrangement in front of the posterior margin, surrounding a larger, less distinct pore, as in the Mediterranean *H. arianii* Wittmann, 2000, and NE. Atlantic *H. dardani* Wittmann, 2008. An additional group with 18–20 pores (Fig. 2C), in strongly flattened ‘V’-shaped arrangement, is in median position, closely in front of cervical sulcus.

***Eyes*** (Figs 1; 2A, B). Thick, shaped in form of dorsoventrally compressed globoids. Cornea appears calotte-shaped to sub-reniform in dorsal and in ventral view, oval in lateral view. Comparatively large group of scales distributed along inner, obliquely anteriorly facing margin of eyestalks. Ocular symphysis with broadly-rounded, smooth, subrostral process (as dashed lines in Fig. 2A, B).

***Antennulae*** (Fig. 2A, B, E). Basal segment 42–47% length of trunk, middle is 12–19% and terminal segment is 36–42%, when measured along dorsal midline of trunk (only Fig. 2E shows entire extension of basal segment). Trunk stouter in males, with basal segment 1.4–1.5 times longer than broad, compared to 1.7–1.8 in females. A small dorsal apophysis and a longer outer ventral lobe (exite) extend (obliquely) forwards from end of basal segment. Dorsal apophysis bears 1–2 smooth setae and a number of barbed setae, ventral lobe bears four plumose setae at its tip. Median segment obliquely truncate, its anterior margin dorsally with a smooth seta in median position and more laterally an additional smooth seta, together with several barbed setae. Two small barbed setae antero-ventrally near outer margin. These last setae not visible in dorsal view (Fig. 2A, B, E). Inner distal corner of terminal segment ventrally with a large, obliquely inwards-forwards directed, plumose seta, this seta larger in females compared to males, and dorsally with an apophysis as described in the diagnosis. In sublateral to submedian position on anterior margin of terminal segment there is a lobe with 3–5 medium-sized barbed setae and a dense series of short bristles. Only females with additional, large plumose setae, also obliquely inwards-forwards directed in Fig. 2B, on terminal segment of trunk, one half-way on inner margin, the second on ventral surface proximally from inner flagellum, at about 25% segment length from anterior margin of terminal segment. Appendix masculina terminally bilobate, forming a medium-sized, forwards directed extension distally on ventral surface of terminal segment, and bearing a large and dense brush of setae extending obliquely downwards (Figs 1; 2A, E). Outer antennular flagellum 1.3–1.5 times as thick as inner flagellum, when measured near basis.

***Antennae*** (Fig. 2A, B). Length of antennal scale 3.2–3.9 times its maximum width; without spines, setose all around. A small apical segment with five plumose setae separated from basal part by an essentially transverse, though slightly-oblique suture; apical segment broader than long, contributing 4–7% to total scale length. Antennal sympod with forwards-projecting tongue-like, terminally rounded lobe; posteriorly with a broad, terminally weakly-bilobate lobe containing end sac of antennal gland. Peduncle three-segmented, clearly shorter than scale. Basal segment 17–22% length of peduncle, second 40–48% and third 34–38%.

***Mouth parts*** (Figs 2F–J, 3A). Labrum, labium, maxillulae and maxillae as normal in this genus. Mandibular palp normal, three-segmented. Median segment with normal setae along inner and outer margins. Pars molaris of both mandibles with strong grinding surface. Pars incisivus with 2–3 large teeth, and digitus mobilis with 2–3 large, plus two small, teeth. Pars centralis (‘spine row’ in terminology of Tattersall and Tattersall 1951) with 5–7 spiny teeth. Distal segment of maxillula terminally with 7–10 strong, inconspicuously serrated spines, subterminally with transverse row of 4–5 weakly barbed setae. Endite of maxillula with two large, distally spinose setae, and total of 12–17 smaller, smooth or barbed setae.

***Thoracopods in general*** (Figs 3B–M; 4A–F; 5A, B). Sizes increase from exopod 1 to 5 or 6 and decrease from 6 to 8. Homologous exopods larger in males than in females. Flagellum of first to eighth exopods with 8, 9, 9, 9, 9, 9, 9, 9 segments in males, or 8, 9, 9, 9, 9, 9, 9, 8 segments in females, respectively, not counting the large intersegmental joint between basis and flagellum. Exopods with basal plate 1.4–2.2 times longer than broad in males, versus 1.8–2.5 in the mostly less broad exopods of females. Lateral expansion distinct in both sexes, outer margin ending in rounded edge. Endopods: for segmental numbers of carpopropodus see diagnosis. Distinct dactylus present in all thoracic endopods. Size of dactylus (Fig. 3E–M) decreases in order of endopod 2 > 1 > 3 > 4 > (5–8). Claws present in endopods 1 and 3–8, but absent in endopod 2. Claws do not differ between sexes.

***Maxillipeds*** (first and second thoracic endopods; Fig. 3B–F). First endopod with sympod bearing large fields of minute hairs, mainly on its outer half. Inner portions of sympod representing basis of endopod. First thoracic epipod large, leaf-like, without setae or hairs, but with small field of minute scales near insertion with sympod (Fig. 3B). Basis of first endopod with short, conical endite ending in one plumose, basally-thick seta. Basis with additional, large prominent endite, ischium and merus with feebly-projecting endites, carpus with again shorter, almost indistinct endite: these endites densely setose on their inner margins. Large endite of basis strongly hairy, that of ischium weakly hairy only at and near its inner margin, that of merus again less hairy in this position, that of carpus not hairy at all. Dactylus with strong, subapically, bilaterally microserrated claw (Fig. 3E). Basis of second endopod with weakly-projecting, subterminally setose endite (Fig. 3C). Merus slender, slightly longer than combined praeischium and ischium, but distinctly shorter than combined carpopropodus and dactylus. Dactylus without claw (Fig. 3F), bearing only a dense brush of setae (Fig. 3C). Among these setae are 9–13 modified setae, each bearing bilateral series of stiff, partly-acute micro-barbs in their subbasal to median portions (Fig. 3D).

***Gnathopods*** (third thoracic endopod; Fig. 4A, B). Endopod weakly powerful, somewhat subchelate. Its carpopropodus in both sexes comparatively slender among species of *Heteromysis*, 4.4–5.7 times longer than broad; length 0.9 times that of merus, and 1.0–1.1 times that of ischium. Modified setae on outer margin of carpus as in diagnosis. Claw smooth, powerful, showing only weak curvature; microserrated in subapical portions of only its outer margin (Fig. 3G). Most setae of endopod smooth or barbed to different degrees.

***Pereiopods*** (fourth to eighth thoracic endopods; Figs 3H–M; 4D–F; 5A, B) all moderately long and slender. Their claws microserrated on two opposite sides of their subapical portions (Fig. 3H–M). Fourth endopod with modified setae on outer margin of its carpopropodus, as in diagnosis. Its moderately small dactylus bearing long, thin, weakly-bent claw (Fig. 3H). Fifth to eighth endopods equipped with again smaller dactylus bearing much shorter claw that shows a stronger, distally-increasing curvature (Fig. 3J–M). Fifth endopod, when stretched, extends well beyond eyes.

***Marsupium.*** Females with large marsupial plates on seventh and eighth thoracopods. Sixth thoracopod with rudimentary oostegite representing a small lobe, on its inner margin with three proximally weakly-barbed setae.

***Penes*** (Fig. 5A). Shape roughly that of rod-like, straight tubes, facing obliquely in anterior direction up to basis of second thoracopod. Each penis very long, stiff, with smooth cuticle; series of three small, barbed setae subterminally on exterior face.

***Pleopods*** (Fig. 4G–J). Length and structure of pleopods do not differ between sexes, with rod-like exopodal portion and shorter lobe-like endopodal portion. The long seta at inner, terminal edge of endopod smooth or almost smooth, all remaining setae well barbed or plumose. Total length of pleopod 5 about twice (198–214%) that of pleopod 1 (n = 4). This increase is not continuous: starting with pleopods 1 versus 2, the length increase between subsequent pleopods is 22–27%, 3–5%, 5–7%, and 41–65%, respectively.

***Uropods*** (Fig. 5C). Exopods reach with 12–21% of their length beyond endopods and 25–33% beyond telson, endopods 15–18% of their length beyond telson. Exopod length 4.1–5.0 times maximum width, inner margin more strongly convex than outer one. Endopods basally with large statocyst, containing discoidal, in dorsal view slightly ellipsoid, statolith of average size. Statoliths with indistinct fundus and distinct tegmen, composed of fluorite; diameter 130–143 µm (n = 4); statolith formula is 2 + 3 + 1 + (7–12) + (7–12) = 21–30. Uropods with densely setose lateral margins, except for their most basal portions.

***Telson*** (Fig. 5D). Length 1.2–1.4 times ultimate abdominal somite, or 0.8–0.9 times exopod of uropods. Length of telson 1.7–1.8 times maximum width. Laminae of cleft show about half average length of lateral spines. Basal half of telson as well as distal portions of its cleft with smooth margins. Lateral spines with almost equal length, their size not increasing distally.

***Colour*** (Fig. 1). General appearance of living specimens light red. Cornea brown-golden; eyestalks mainly light red, except for a light spot near inner anterior corner and a narrow white ribbon along posterior, dorsal portions of the inner margin of the cornea (best visible in left eye of upper male in Fig. 1B). Red chromatophore centres scattered over eyestalks, antennae, carapace, pleon, uropods, and telson. Transverse double series of chromatophores near posterior margin of each thoracomere 8 and pleomere 1-6; additional chromatophores on pleomere 6. Uropods with chromatophores over entire length. Telson with the greatest density of chromatophores. These colours disappeared within a few weeks of fixation, except for some dark brown pigment in cornea. White ribbon on eyestalks persisted longer than red colour on remaining parts of stalk.

***Nauplioid stage*** (Fig. 3N). Breeding female of 6.4 mm body length carried three nauplioid larvae at late substage 2, length 1.2–1.3 mm (n = 3). The female of 6.1 mm showed 11 naupliods at substage 3, length 1.0–1.1 mm (n = 8). Besides features typical of the respective state of development, one finds small setae near tip of antennulae and antennae and a pair of cercopods flanking end of larval abdomen. Each cercopod bears 10–15 acute spines with apically-increasing size (n = 11). A number of additional very small spines (or bristles) present on terminal tip of body, a few also more anteriorly on ventral face of larval abdomen.

##### Etymology.

The species name is a noun in genitive singular, adopted from the hermit crab host *Cancellus*.

##### Type locality.

Sublittoral marine coastal waters at Roman Rock, off Simonstown, on the False Bay coastline of the Cape Peninsula, South Africa, 34°10.95'S, 18°27.50'E, 20 m depth. *H. cancelli* sp. n. was only found in a single gastropod shell inhabited by the hermit *Cancellus macrothrix* Stebbing, 1924, although four other *Cancellus* specimens and numerous other hermit crabs, mainly *Paguristes gamianus* (H. Milne-Edwards, 1836), were collected and examined from this and nearby sites.

#### Heteromysis (Heteromysis) fosteri

Taxon classificationAnimaliaMysidaMysidae

Wittmann & Griffiths sp. n.

http://zoobank.org/15D6AFA7-B148-4474-B348-C7F011A0D3E7

Figs 6A , 7 , 8

##### Type series.

Holotype, adult male with 6.7 mm body length, in vial at NHMW-25905; paratypes adult female 7.0 mm, adult male 6.6 mm, 2 subadult males 3.6, 4.6 mm in vial at SAM-MB-A067551; dissected paratypes on slides at NHMW-25906: adult female 8.0 mm, subadult female 5.9 mm, adult male 6.0 mm; sublittoral marine coastal waters, Miller’s Point, on the False Bay coastline of the Cape Peninsula, South Africa, 34°13.78'S, 18°28.47'E, 5 m depth; extracted from empty urchin and gastropod shells without hermit crabs, 25 Jan. 2017, leg. Craig Foster.

##### Diagnosis.

Carapace produced into well-projecting, triangular rostrum with narrow rounded apex. Eyes well developed; cornea occupies 35–50% of eye surface; eyestalks with inconspicuous, distally-directed, blunt extension of (obliquely anterior facing) inner margin. Antennular trunk with a number of smooth and barbed setae, but no particularly modified setae; inner distal corner of its terminal segment with anteriorly directed apophysis carrying two large, smooth setae. Antennal scale stout, extending to 0-10% below tip of antennular trunk; outer margin convex. First thoracic sternite with anteriorly-projecting, terminally-rounded median lobe; sternites 2–8 without lobes in both sexes. Carpopropodus of thoracic endopods 1–8 with 2, 2, 2, 3, 6, 6, 6, or 5-6 segments, respectively. Third thoracic endopod weakly dimorphic, without any spines or spine-like setae; carpus not swollen in female, slightly swollen in male (with respect to merus). Carpus of third thoracic endopod with series of three subbasally toothed setae (i.e. with modified barbs) near outer margin. Carpopropodus of fourth thoracic endopod with series of 2–3 subbasally toothed setae near outer margin; no such modified setae in endopods 5–8. Penes long and slender, twice length of merus of eighth thoracic endopod; tip with three rounded lobes, each wider than long. Pleopods reduced to small setose, bilobate plates, without any spines in both sexes. Exopods of uropods extend distinctly beyond endopods. Endopods with only one spine on inner margin, in subbasal position near statocyst. Telson subtriangular, terminally truncate; lateral margins weakly-sigmoid, along their distal 83–93% furnished with slightly discontinuous series of 17–20 spines each. Telson with apical cleft forming proximally poorly rounded ‘V’. This cleft slightly deeper than wide, its depth 14–18% telson length. Cleft densely furnished with 16–24 acute laminae all along its margins. Two latero-apical lobes of telson show narrow, transverse apical margins, each bearing a large latero-apical spine plus much smaller medio-apical spine.

##### Description.

As described above for *H. cancelli* sp. n. unless stated otherwise in the following. Cephalothorax comprises 41–44% of body length without telson, pleon 56–59%, and carapace 30–38%, when measured along dorsal median line. First thoracic sternite with median lobe showing a smooth, rounded apex. Terminal margin of sixth pleonite with lateral shields more evenly rounded in females (Fig. 7L) versus males (Fig. 7K).

**Figure 6.**
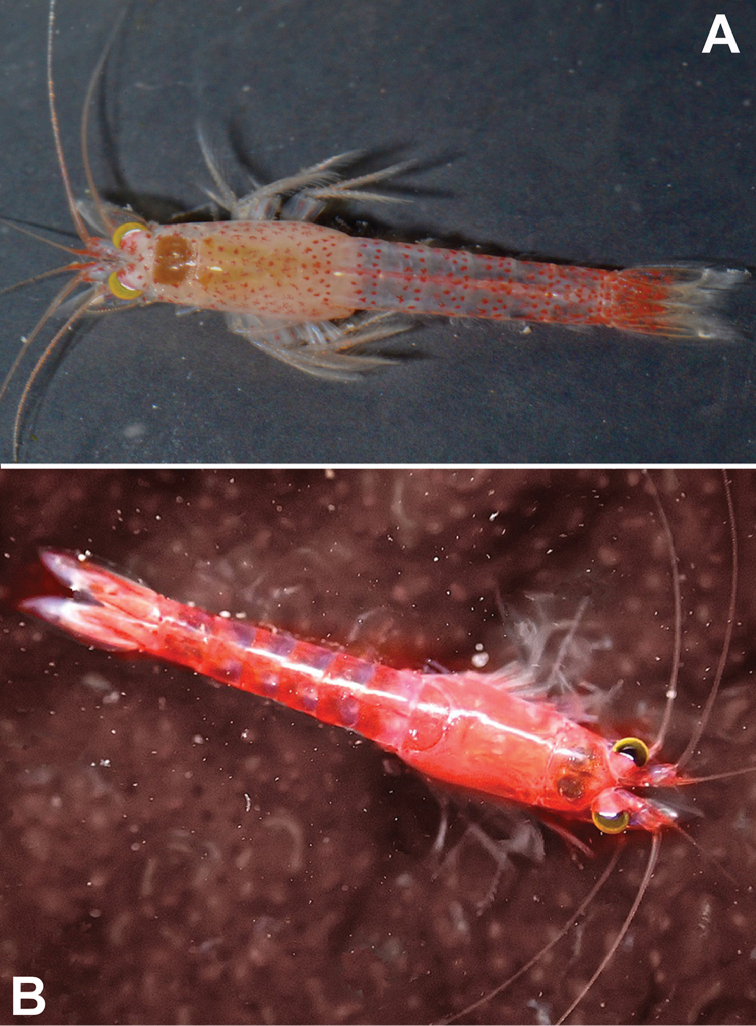
Microphotographs of adult female of *Heteromysis fosteri* sp. n. with 7 mm body length (**A**) and of adult male of *Heteromysis octopodis* sp. n. with 9 mm (**B**), each from Miller’s Point, Cape Peninsula, South Africa. **A** laboratory photo by C.L. Griffiths **B** in situ image by Craig Foster, this last with background cleaned with electronic tools.

**Figure 7.**
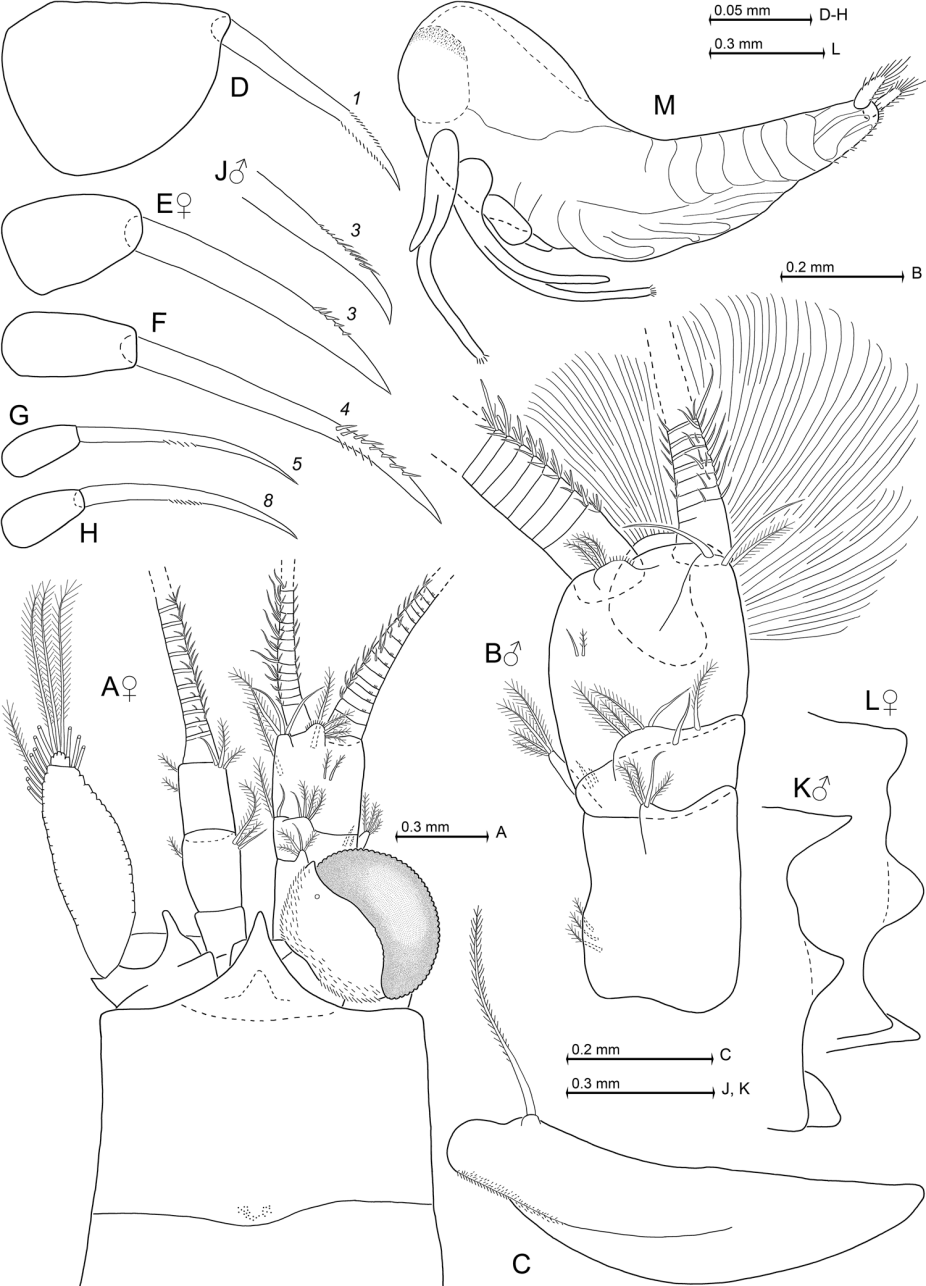
*Heteromysis fosteri* sp. n., paratype females with 7.0 mm (**A**) and 8.0 mm (**C–H, L**) body length, paratype male 6.0 mm (**B, J, K**). **A** cephalic region plus anterior part of carapace in female, dorsal view **B** male antennula, dorsal **C** epipod of first thoracopod, caudal **D–H** series of dactylus with claw in thoracic endopods 1, 3-5, 8 of female, caudal **J** tip of claw of third thoracic endopod in male **K, L** terminal margin of sixth pleonite, lateral, in male (**K**) versus female (**L**) **M** nauplioid larva at substage 3, lateral.

***Carapace*** (Figs 6A, 7A). Non-dimorphic, antero-lateral edges evenly rounded. Anterior pore group with 25–34 pores, in strongly flattened ‘U’-shaped arrangement. Posterior pore group with 12–16 pores.

***Eyes*** (Figs 6A, 7A). Anterior, basal, and posterior margins of eyestalks densely covered by scales. Ocular symphysis with broadly-rounded, smooth, subrostral process (dashed line in Fig. 7A).

***Antennulae*** (Fig. 7A, B). Basal segment 41–48% the length of trunk, middle 14–18% and terminal segment 37–43%, when measured along dorsal midline of trunk. Trunk stouter in males, with basal segment 1.1–1.2 times longer than broad, compared to 1.3 times in females. Basal segment with three barbed and one smooth seta at its dorsal apophysis; four plumose setae at tip of outer ventral lobe (exite). Anterior margin of median segment dorsally with a smooth seta and larger barbed seta; and more laterally another smooth seta together with three barbed setae in both sexes. Two small barbed setae antero-ventrally near outer margin, visualized by dashed lines in Fig. 7B. Anterior margin of terminal segment in about median position with a lobe bearing 3–4 medium-sized barbed setae plus a dense series of short bristles. Only females with additional, large plumose setae on terminal segment of trunk, one inserting ventrally half-way near inner margin, the second, forward directed in Fig. 7A, on the ventral surface proximally from the inner flagellum at about 9–19% segment length from anterior margin of terminal segment. Outer antennular flagellum 1.5–1.7 times as thick as inner flagellum, when measured near basis.

***Antennae*** (Fig. 7A). Length of antennal scale 2.7–3.0 times its maximum width. Basal segment 24–29% length of peduncle, second 33–39% and third 34–38%.

***Mouth parts*** (not figured). Pars incisivus of mandibles with 3–4 large teeth, and digitus mobilis with 3–4 large plus 1–3 small teeth. Pars centralis with 4–5 spiny teeth. Distal segment of maxillula terminally with 8–10 strong, inconspicuously serrated spines, subterminally with a transverse row of 5–6 barbed setae. Endite of maxillula with three large, distally-spinose setae, and a total of 15–20 smaller, smooth or barbed setae.

**Figure 8.**
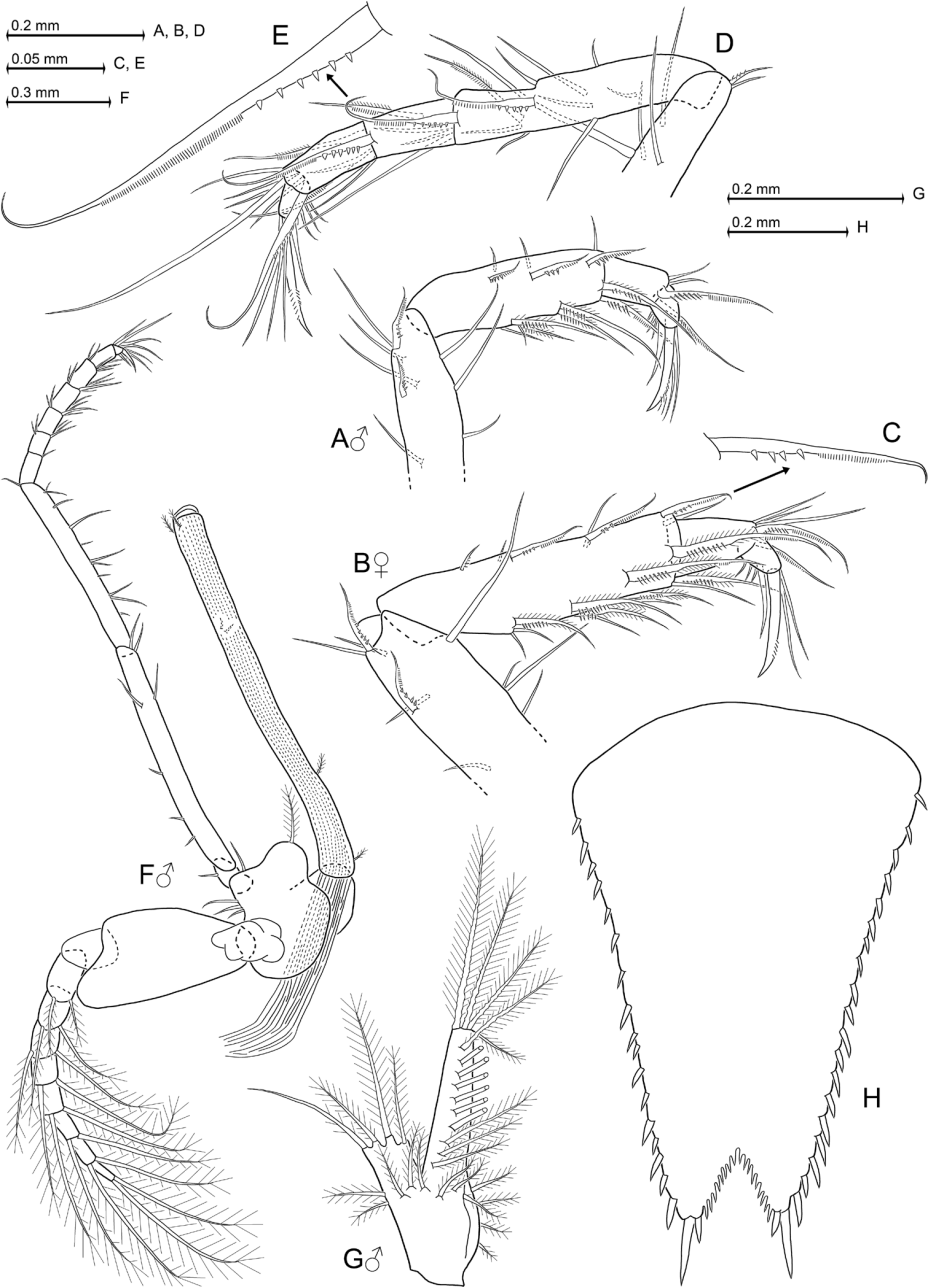
*Heteromysis fosteri* sp. n., paratype male (**A, F–H**) with 6.0 mm body length and paratype female 8.0 mm (**B–E**). **A** male tarsus with part of merus pertaining to third thoracic endopod, caudal aspect **B** the same for female, detail (**C**) shows subbasally toothed seta **D** tarsus of fourth thoracic endopod, caudal, detail (**E**) shows subbasally toothed seta **F** eighth thoracopod with penis, caudal **G** third male pleopod, rostral **H** telson, dorsal.

***Thoracopods in general*** (Figs 7D–J, 8A–F). Sizes increase from exopod 1 to 4 and then show no clear trend from 4 to 8. Homologous exopods are not clearly different between sexes. Flagellum of first to eighth exopods with 8, 9, 9, 9, 9, 9, 9, 8–9 segments. Exopods with basal plate 1.4–2.0 times longer than broad in both sexes. Claw of third endopod more strongly serrated in males (Fig. 7J) versus females (Fig. 7E), to a minor degree also in fourth endopod, not so in remaining endopods.

***Maxillipeds*** (first and second thoracic endopods; Fig. 7C, D). First endopod with hairs on outer half of sympod. Its epipod large, leaf-like, with comparatively long field of minute scales near insertion with sympod, and subbasally with a large seta densely barbed along its distal 60–75%. Dactylus of second endopod with dense brush of setae, among these 10–15 modified ones.

***Gnathopods*** (Figs 7E, J; 8A–C). Carpopropodus of endopod comparatively slender, four times longer than broad in males, versus five times in females; length is 0.8–0.9 times that of merus and 0.9–1.0 times that of ischium in both sexes. Subbasally toothed setae (Fig. 8C): three on carpus plus two in (sub)apical position near outer margin on merus (Fig. 8A, B).

***Pereiopods*** (Figs 7F–H, 8D–F). Carpus of fourth endopod (Fig. 8D, E) with longer, subbasally toothed setae compared to third endopod (Fig. 8A–C). Fourth endopod with moderately small dactylus bearing long, strong, weakly-bent claw, microserrated on two opposite sides of subapical portions (Fig. 7F). Fifth to eighth endopods equipped with again smaller dactylus bearing a much shorter claw that shows slightly stronger, distally-increasing curvature; this claw unilaterally microserrated only in median portions of its inner margin (Fig. 7G, H). Sixth thoracopod of females with rudimentary oostegite representing a small lobe, on its inner margin with 0–2 proximally weakly barbed setae.

***Penes*** (Fig. 8F) as above in diagnosis. Presence of small, barbed setae: 1–2 subterminally on exterior face plus 4–5 ones scattered anywhere between 3% and 70% penis length from basis.

***Pleopods*** (Fig. 8G). The seta at inner terminal edge of endopod shows only minor number of barbs, all remaining setae well-barbed or plumose. Total length of pleopod 5 is 177–190% that of pleopod 1 (n = 4). Starting with pleopods 1 versus 2, length increase between subsequent pleopods is 19–32%, 0–13%, 1–18%, and 22–33%, respectively.

***Uropods*** (not figured). The exopods reach with 21–30% of their length beyond endopods and 17–25% beyond telson, the endopods 6–11% of their length beyond telson. Exopod length 4.3–4.4 times maximum width. Statoliths composed of fluorite; diameter 145–181 µm (n = 4); statolith formula is 2 + 3 + 1 + (8–13) + (16–19) = 31–38.

***Telson*** (Fig. 8H). Length 1.2 times that of ultimate abdominal somite or 0.7–0.8 times exopod of uropods. Length of telson 1.2–1.5 times its maximum width. Laminae of cleft show 0.6–0.8 times average length of lateral spines. Length of lateral spines distally discontinuously increasing in size by a factor of about 1.6, arming almost all outer margin.

***Colour*** (Fig. 6A). General appearance of living specimens light red with orange tinge. The stronger orange tinge of the thorax in Fig. 6A is due to yolk in the ovarian tubes. Cornea yellow-golden; eyestalks mainly light red, except for a white ribbon along posterior, dorsal portions of the inner margin of the cornea. Red chromatophore centres scattered over eyestalks, antennae, pleon, uropods, and most densely over carapace, thoracomere 8, and telson. Two transverse series of chromatophores, one in about the middle and the second near posterior margin of pleomeres 1–2, and less regularly arranged also on pleomeres 3–6. Starting with pleomere 3, additional chromatophores are scattered with increasing density up to pleomere 6. Uropods with chromatophores only over proximal 70%. Persistence of colours as in *H. cancelli* sp. n.

***Nauplioid stage*** (Fig. 7M). One female with body length 8.0 mm carried 13 nauplioid larvae at substage 3, length 1.1–1.3 mm. At that same state of development one finds the same external morphological features as in *H. cancelli* sp. n. (Fig. 3N shows substage 2). Each cercopod bears 10–14 acute spines with apically increasing size.

##### Etymology.

The species name is a noun in genitive singular, named after well-know underwater-photographer and diver Craig Foster, who has accompanied and assisted the second author in many collecting expeditions and who made all in situ photographs and sampled all specimens of both this species and the one following.

##### Type locality.

Sublittoral marine coastal waters at Miller’s Point, on the False Bay coastline of the Cape Peninsula, South Africa, 34°13.78'S, 18°28.47'E. This is only 5.5 km to the SSE of the type locality of *H. cancelli* sp. n. (above). Materials were extracted from mixed collection of dead shells mainly belonging to the whelk *Argobuccinum pustulosum* (Lightfood, 1786), the winkle *Turbo cidaris* Gmelin, 1791, and the urchin *Parechinus angulosus* (Leske, 1778).

#### Heteromysis (Heteromysis) octopodis

Taxon classificationAnimaliaMysidaMysidae

Wittmann & Griffiths sp. n.

http://zoobank.org/6C36EF49-CECB-4506-BD82-0B49ACCBD83B

**Figure 9.**
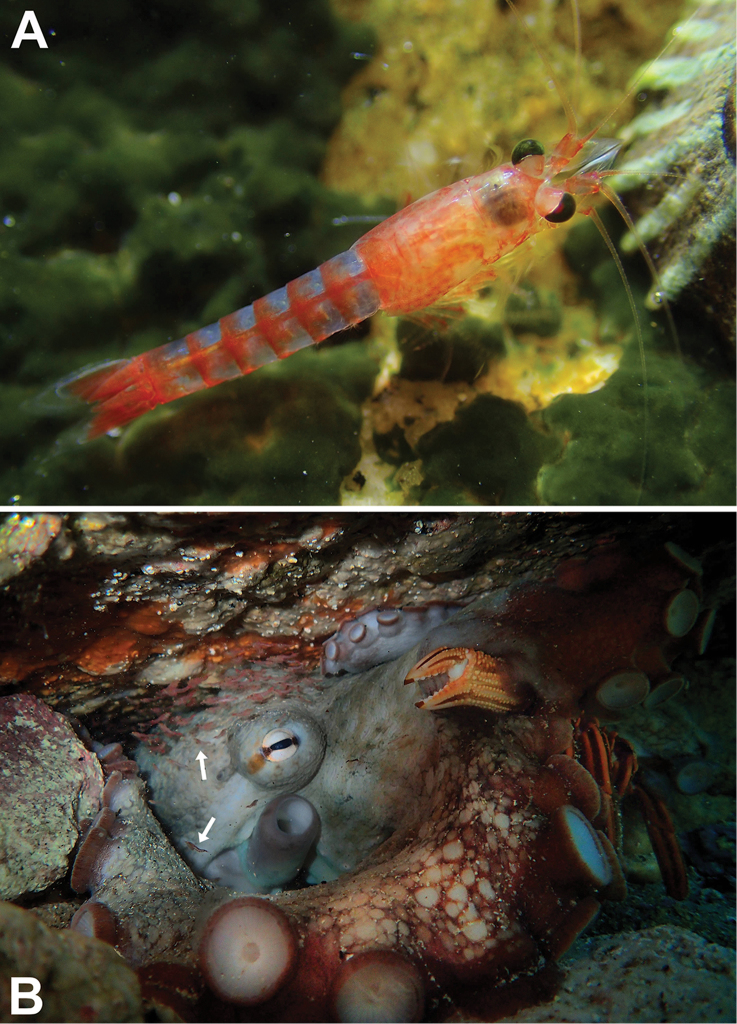
**A** subadult female of *Heteromysis octopodis* sp. n. with 11 mm body length from tide pool **B** multi-species association inside den in 3 m depth, occupied by *Octopus vulgaris*, to the right with the crab *Guinusia chabrus*; upper arrow points to a mysid school of what we assume to be *H. octopodis* sp. n., lower arrow to a different but undetermined mysid species. **A, B** from Miller’s Point, Cape Peninsula, South Africa; in situ images by Craig Foster **B** image is taken of the same octopus den from which the samples were collected, but on a different date.

**Figure 10.**
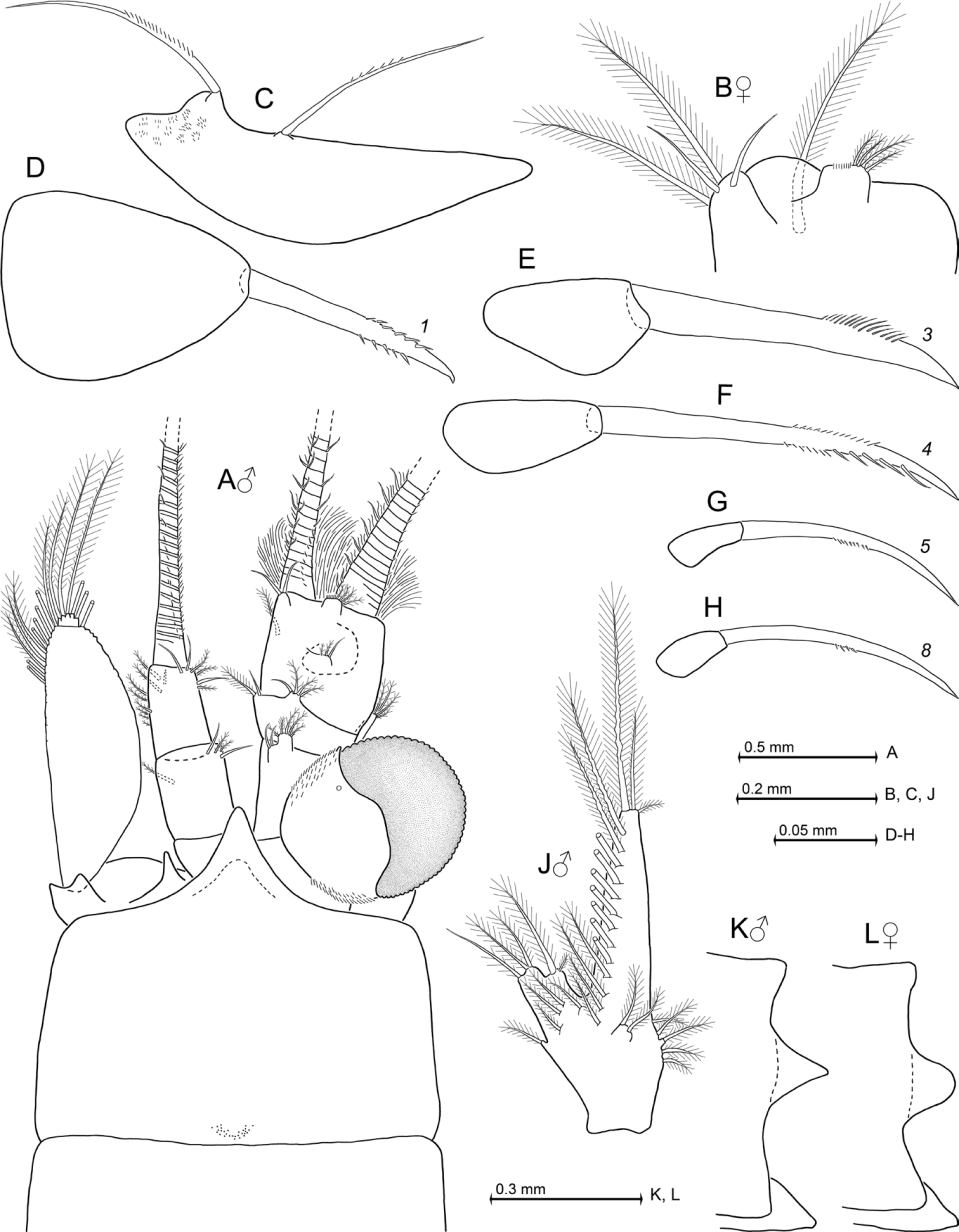
*Heteromysis octopodis* sp. n., paratype adult male with 8.8 mm body length (**A, D–K**), paratype subadult females with 9.0 mm (**B, L**) and 8.8 mm (**C**). **A** cephalic region plus anterior part of carapace in male, dorsal view **B** anterior margin of antennular trunk in subadult female, dorsal **C** epipod of first thoracopod, caudal **D–H** series of dactylus with claw in thoracic endopods 1, 3-5, 8, caudal **J** fourth male pleopod, rostral **K, L** terminal margin of sixth pleonite, lateral, in male (**K**) versus subadult female (**L**).

**Figure 11.**
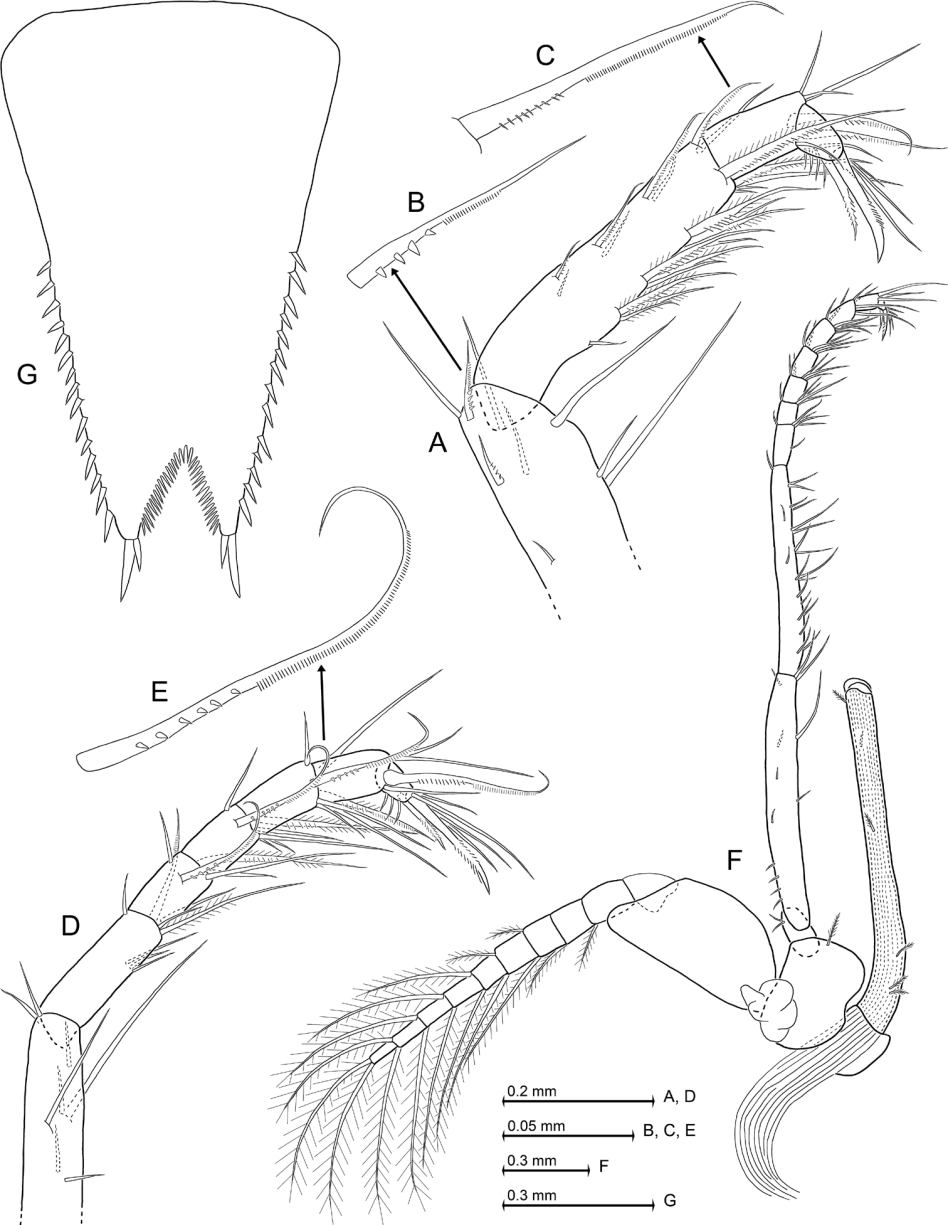
*Heteromysis octopodis* sp. n., paratype adult male with 8.8 mm body length. **A** tarsus with part of merus pertaining to third thoracic endopod, caudal aspect, details show subbasally toothed seta on merus (**B**) versus subbasally barbed seta on carpus (**C**) **D** tarsus with part of merus pertaining to fourth thoracic endopod, caudal, detail (**E**) shows subbasally toothed seta **F** eighth thoracopod with penis, caudal **G** telson, dorsal.

Figs 6B , 9 , 10 , 11

##### Type series.

All materials from Miller’s Point, on the False Bay coastline of the Cape Peninsula, South Africa, leg. Craig Foster. Holotype, adult male with 7.0 mm body length, in vial at NHMW-25907; paratypes immature female 6.2 mm and immature male 6.1 mm, in vial at SAM-MB-A067552; dissected paratypes adult male 6.8 mm, subadult female 10.8 mm, immature female 7.8 mm, on slides at NHMW-25908, associated with *Octopus vulgaris* inside den in 3 m depth, 34°13.77'S, 18°28.45'E, 15°C, 26 March 2017; paratype adult male 8.8 mm (dissected, slides at NHMW-25909) from euhaline intertidal pool, 34°13.79'S, 18°28.43'E, 10 Aug. 2014; paratypes 2 subadult females (9.4 mm entire, 9.0 mm in 2 parts, both in vial at SAM-MB-A067553) plus damaged subadult female (estimated original size 8.8 mm, head missing, dissected, slides at NHMW-25910), from same pool as before, at a few cm depth upon low tide, 8 Sept. 2015.

##### Diagnosis.

Based only on adult males and subadult females. Carapace produced into well-projecting, (sub)triangular rostrum with rounded apex. Eyes well-developed; cornea occupies 60–70% of eye surface; eyestalks with inconspicuous, distally-directed, blunt extension of (obliquely anterior facing) inner margin. Antennular trunk with a number of smooth and barbed setae, but no particularly modified setae; inner distal corner of its terminal segment with apophysis carrying two medium-sized, smooth setae. Antennal scale stout, extending to 0–20% below tip of antennular trunk; outer margin convex. First thoracic sternite with anteriorly-projecting, terminally-rounded median lobe; sternites 2-8 without lobes. Carpopropodus of thoracic endopods 1-8 with 2, 2, 2-3, 4-6, 5-7, 5-8, 6-8, or 6-8 segments, respectively. Third thoracic endopod without any spines or spine-like setae; carpus not swollen with respect to merus. Carpus with series of three weakly-modified setae near outer margin; these setae subbasally furnished with bristles. Merus (sub)terminally with two subbasally toothed setae. Fourth thoracic endopod with series of 2–3 such toothed setae near outer margin of carpopropodus; no such modified setae in endopods 5–8. Penes long and slender, 1.6–1.7 times length of merus of eighth thoracic endopod; tip with three rounded lobes, each wider than long. Pleopods reduced to small setose, bilobate plates, without any spines. Exopods of uropods extend distinctly beyond endopods. Endopods with only one spine on inner margin, in subbasal position near statocyst. Telson subtriangular, terminally truncate; lateral margins weakly sigmoid, along their distal 46–53% furnished with almost continuous series of 13–17 spines each. Telson with apical cleft forming a proximally rounded ‘V’. Cleft slightly deeper than wide, its depth 17–23% telson length. Cleft densely furnished with a total of 26–37 acute laminae all along its margins. Two latero-apical lobes of telson show narrow transverse apical margins, each bearing a large latero-apical spine, plus much smaller medio-apical spine.

##### Description.

As described above for *H. cancelli* sp. n. unless stated otherwise in the following. Cephalothorax comprises 37–44% of body length without telson, pleon 54–63%, and carapace 32–38%, when measured along dorsal median line. First thoracic sternite with median lobe showing smooth rounded apex. Each of first to fifth abdominal somites measure 0.8–1.1 times length of sixth somite. Terminal margin of sixth pleonite with lateral shields triangular, with tip more rounded in subadult females (Fig. 10L) than in males (Fig. 10K).

***Carapace*** (Figs 6B, 9, 10A). Non-dimorphic, antero-lateral edges evenly rounded. Cervical sulcus well marked, cardial sulcus weak but distinct. Anterior pore group of carapace with 29–32 pores in strongly flattened ‘U’-shaped arrangement; posterior group with 12–15 pores (n = 6).

***Eyes*** (Figs 6B, 9, 10A). Anterior and posterior margins of eyestalks densely covered by scales, but not so the basal portions. Ocular symphysis with subtriangular to sinusoid, in any case terminally rounded, smooth, subrostral process (dashed line in Fig. 10A).

***Antennulae*** (Fig. 10A, B). Basal segment 41–49% length of trunk, middle 14–18% and terminal segment 34–44%. Trunk on the average stouter in males, with basal segment 0.9–1.4 times longer than broad, compared to 1.2–1.5 times in subadult females. Terminal portion of basal segment with bifid dorsal apophysis bearing 4–5 barbed setae on its more median projection, plus two smooth setae on its outer projection. Outer ventral lobe of basal segment bears four plumose setae at its tip plus a small barbed seta in subbasal position. Anterior margin of median segment dorsally with apophysis bearing a smooth seta together with several barbed setae; inner margin anteriorly with medium-sized to large barbed seta together with smooth seta. Two small barbed setae antero-ventrally close to outer margin (not visible in Fig. 10A). Terminal segment ventrally with 1–2 large, obliquely forwards-directed, plumose setae and 0–2 additional plumose setae dorsally at inner distal corner in both sexes. Lobe with four medium-sized barbed setae plus a dense series of short bristles in about median position on anterior margin of terminal segment. Outer antennular flagellum 1.3–1.5 times as thick as inner flagellum, when measured near basis.

***Antennae*** (Fig. 10A). Length of antennal scale 2.8–3.2 times its maximum width. Apical segment contributing 5–7% to total scale length. Basal segment 21–28% length of peduncle, second 36–40% and third 34–40%.

***Mouth parts*** (not figured). Pars incisivus of mandible with 3–4 large teeth, and digitus mobilis with 3–4 large plus 2–3 small teeth. Pars centralis with 3–4 spiny teeth. Distal segment of maxillula terminally with 9–12 weakly serrated spines, subterminally with a transverse row of 5–6 barbed setae. Endite of maxillula with three large, distally spinose setae, and 18–28 smaller, smooth or barbed setae.

***Thoracopods in general*** (Figs 10D–H; 11A–F). Sizes increase from exopod 1 to (4–6) and decrease from 6 to 8. Flagellum of first to eighth exopods with 8, 9, 9, 9, 9, 9, 9, 9 segments. Exopods with basal plate 1.3–2.2 times longer than broad. Claws of endopods 1, 3, 4 more strongly serrated in male versus subadult female, not so in endopods 5–8.

***Maxillipeds*** (Fig. 10C, D). Sympod of first endopod with hairs on outer half. First thoracic epipod large, leaf-like, with small field of minute scales near insertion with sympod, and (sub)basally with 1–2 large, sparsely barbed setae (Fig. 10C). Dactylus of first endopod with strong, subapically, bilaterally-microserrated claw (Fig. 10D). Dactylus of second endopod with dense brush of setae, among these 16–19 modified ones.

***Gnathopods*** (Figs 10E, 11A–C). Endopod with comparatively slender carpopropodus, 4.9–5.5 times longer than broad; length 0.8–1.0 times that of merus, and 0.9–1.0 times that of ischium.

***Pereiopods*** (Figs 10F–H, 11D–F). Fourth endopod with moderately-small dactylus bearing a long, weakly-bent claw, microserrated on two opposite sides of its subapical portions (Fig. 10F). Fifth to eighth endopods equipped with again smaller dactylus bearing much shorter claw that shows a stronger, distally increasing curvature; this claw unilaterally microserrated only in median portions of inner margin (Fig. 10G, H).

***Penes*** (Fig. 11F) long, facing obliquely in anterior direction up to basis of fourth thoracopod. Five to six small, barbed setae scattered all along the penis.

***Oostegites.*** The subadult females already have eggs in the ovaries, visible in Fig. 9A by yellow complexion through the semi-transparent, essentially red carapace (best visible at the rostrum). The subadults show well-formed but still immature marsupial plates on the seventh and eighth thoracopods, plus rudimentary oostegites on the sixth thoracopods.

***Pleopods*** (Fig. 10J). The seta at inner, terminal edge of endopod weakly barbed, remaining setae well-barbed or plumose. Part of plumose setae of exopod with wave-like series of weak constrictions along shaft. Total length of pleopod 5 is 173–207% that of pleopod 1 (n = 7). Starting with pleopods 1 versus 2, the length increase between subsequent pleopods is 14–31%, 3–12%, 0–15%, and 21–58%, respectively.

***Uropods.*** The exopods reach with 15–38% of their length beyond endopods and 33–43% beyond telson, endopods 6–17% of their length beyond telson. Exopod length 3.8–4.2 times maximum width. Statoliths composed of fluorite; diameter 120–196 µm (n = 12); statolith formula 2 + 3 + (0–1) + (8–16) + (12–19) = 27–39.

***Telson*** (Fig. 11G). Length 1.2–1.5 times that of ultimate abdominal somite, or 0.7–0.9 times exopod of uropods. Length of telson 1.5–1.6 times its maximum width. Laminae of cleft show about 0.5–0.7 times average length of lateral spines. Basal half of outer margins smooth. Length of lateral spines distally (somewhat discontinuously) increasing in size by a factor of 1.3–1.7 .

***Colour*** (Figs 6B, 9). General appearance of living specimens red-orange to blazing red. Cornea brown to yellow-golden; eyestalks red, except for a white ribbon along posterior, dorsal portions of the inner margin of the cornea (best visible in Fig. 6B). Chromatophores could only partly be discerned individually due to their strong expansion (stronger in Fig. 6B than in Fig. 9A) in three specimens micro-photographed alive. Red spots scattered over eyestalks, antennae, carapace, pleon, uropods, and telson. Transverse double series of spots near posterior margin of each pleomere, not well distinguishable in thoracomeres; a broader posterior red band on pleomere 6. Uropods and telson most intensively red. Compared with that of the male in Fig. 6B, the more orange tinge of the female thorax in Fig. 9A comes from yellow yolk in the ovarian tubes. Persistence of colours as in *H. cancelli*.

##### Etymology.

The species name *octopodis* is a noun in genitive singular, derived from the substantive octopus by using the third declension of New Latin.

##### Type locality.

Octopus den (Fig. 9B) in 3 m depth at Miller’s Point, on the False Bay coastline of the Cape Peninsula, South Africa, 34°13.79'S, 18°28.43'E. This is closely adjacent to the type locality of *H. fosteri* sp. n. (above).

##### Microdistribution.

Schools of this mysid were encountered during daytime in shallow sublittoral waters inside dens occupied by *Octopus vulgaris* (in case of Fig. 9B together with a crab and an additional, undetermined mysid species). Association with octopus appears a regular phenomenon as *H. octopodis* was found there nine times between 14 May 2016 and 26 March 2017. Nonetheless, this mysid species was also found upon low tide in a few cm depth in rocky tide pools (Figs 6B, 9A). Also this kind of microdistribution appears to be a regular phenomenon since it occurred there upon various excursions, namely 10 Aug. 2014, 14 Aug. 2014, and 8 Sept. 2015.

## Discussion

### Relationships of the new species

There is a close morphological relationship between the three new *Heteromysis* species from the Cape Peninsula, which share most details described above for the eyes, antennula, mouth parts, carapace, and uropods. This regards also colour as the new species are essentially red, but share a white stripe along the dorso-lateral terminal margin of the eyestalks. As major differences from *H. cancelli* sp. n., *H. fosteri* sp. n. shows relatively shorter telson cleft, different distribution of laminae within the telson cleft, shorter spine-free basal portion of the lateral margins of the telson, as well as differently modified setae on merus and carpus of thoracic endopod 3 and on carpopropodus of endopod 4. *H. octopodis* sp. n. is most similar to *H. fosteri*, but is distinguished mainly by a longer spine-free basal portion of the lateral margins of the telson, by more segments at the carpopropodus of thoracic endopod 4, and by differently modified setae on carpus of endopod 3. It differs from *H. cancelli* by relatively shorter telson cleft, different distribution of laminae within the telson cleft, and by more segments together with differently modified setae on carpopropodus of endopod 4. Both, *H. forsteri* and *H. octopodis*, differ from *H. cancelli* by endopod 4 with shorter and less slender claw, and by endopods 5–8 with claws serrated on only their inner margin, while the outer margin is smooth.

### Subgeneric assignment

The three new species described here are concordant with the original definition of the subgenus Heteromysis S.I. Smith, 1873, by non-dimorphic, rudimentary pleopods without spines or spine-setae. Among the eleven species of the genus *Heteromysis* listed for the E. Atlantic in the key below, six belong to this subgenus. Three species show dimorphic pleopods together with a subapically flagellate, modified seta near the inner distal corner of the antennular trunk, typical of the subgenus Olivemysis Băcescu, 1968, according to the diagnosis of Price and Heard (2011). For two species the available data do not suffice for subgeneric assignment.

Among the three previously described E. Atlantic species of the subgenus Heteromysis, only H. (Heteromysis) microps (G.O. Sars, 1877) shares a single spine on the endopods of uropods with the three additional, new species described here for this subgenus. However, *H. microps* differs clearly from each new species by eyes with smaller cornea, by median sternal processes present on the last five thoracic somites, and by a single spine on basal third of each lateral margin of the telson. Single spines on the endopods of uropods are also found outside the E. Atlantic (two species without subgeneric assignment): in *H. nouveli* Brattegard, 1969, and H. (Heteromysis) spottei Price & Heard, 2000, from W. Atlantic; H. (Heteromysis) australica Băcescu & Bruce, 1980, H. (Olivemysis) kushimotensis Murano & Fukuoka, 2003, H. (Olivemysis) meenakshiae Bamber, 2000, and H. (Olivemysis) panamaensis O.S. Tattersall, 1967, from Pacific; and *H. proxima* W.M. Tattersall, 1922, from Indian Ocean. One to four spines are found in H. (Olivemysis) mayana Brattegard, 1970, from W. Atlantic. The three new species differ from members of the subgenus Olivemysis by absence of flagellate, modified setae on antennular trunk and by male pleopods without spines (spine-setae); and from the remaining five species listed above, including *H. microps*, by absence of spines or spine-like setae on carpopropodus of third thoracic endopod.

### Larval morphology

The nauplioids of *Heteromysis cancelli* sp. n. and *H. fosteri* sp. n. share setae at the tip of antennulae and antennae, so far known, with certain other species of Heteromysini, for example with *H. wirtzi* Wittmann, 2008, *H. ekamako* Wittmann & Chevaldonne, 2016, and *Ischiomysis peterwirtzi* Wittmann, 2013. Among these five species, *H. cancelli* and *H. fosteri* are the only ones showing cercopods close to the tip of the nauplioid abdomen. Including the new findings for *Heteromysis* (Figs 3N, 7M) cercopods or furcal spines, respectively, are currently known from diverse species belonging to only eight genera within the Mysida, all genera pertaining to the family Mysidae: *Acanthomysis*, *Hemimysis*, *Heteromysis*, *Metamysidopsis*, *Mysidium*, *Mysis*, *Neomysis*, and *Praunus* (Nusbaum 1887, Bergh 1893, Manton 1928, Davis 1966, Green 1970, Modlin 1979, Wittmann 1981, Carreño et al. 1989, Jirikowski et al. 2015; taxonomy in part updated, data in part derived from drawings). These genera belong to the subfamilies Heteromysinae, Leptomysinae, and Mysinae, respectively, while no caudal furcae are so far known from at least six out of the ten currently acknowledged subfamilies of the Mysidae. The situation is not clear for Boreomysinae, where Jepsen (1965) found “polyspinal appendages” on the abdomen of nauplioids of *Boreomysis arctica* (Krøyer, 1861), however in much more anterior position compared to normal caudal furcae. A caudal furca is well known from ‘lower’ Crustacea, according to Wittmann et al. (2014) suggesting that the presence of a furca found in nauplioid larvae of a limited number of Mysidae, represents a case of plesiomorphism.

### Microhabitat

As in most species of *Heteromysis*, the three new species show a strictly benthic mode of live. The occurrence of *H. fosteri* in empty shells is in line with other five *Heteromysis* species reported by San Vicente and Monniot (2014) from this and mostly also from other free-living habitats. A major number of species hovers in cryptic habitats during the day (may emerge during the night), such as dense vegetation, gravel, empty shells, coralloid habitats, microcaves or larger marine caves.

As for *H. cancelli* and *H. octopodis*, benthic invertebrates may serve for many species as cryptic habitats in a broader sense, such as sponges, stony corals, gorgonians, sea anemones, brittle stars, tubes of annelids, and shells inhabited by hermit crabs (see list in Fukuoka 2005). The occurrence of *H. octopodis* in tide pools is shared with only few species of *Heteromysis* (Tattersall and Tattersall 1951, Bousfield 1958, Băcescu 1986, Fukuoka 2005). Such microdistribution regards mainly a minor fraction within or among populations as in the well known E. Atlantic and Mediterranean species *Heteromysis norvegica* G.O. Sars, 1883, that hides in tide pools under stones at low tide. It may shelter also in gastropod shells or in dense vegetation. During daytime it is (epi)-benthic in 0-400 m depth (Lagardère and Nouvel 1980, Frutos and Sorbe 2013) and may be planktonic during the night (Tattersall and Tattersall 1951: reported as *H. formosa* S.I. Smith, 1874). Shallow water species of diverse other mysid genera are often found in rocky tide pools or among weed at low tide (Tattersall and Tattersall 1951). Intertidal distribution as a component of normal life habit is mainly found in species of the subfamily Gastrosaccinae, which are sand-burrowing during low tide and/or during daytime and may emerge into the water column during the night (Takahashi and Kawaguchi 1997).

### Commensalism with invertebrates

The facultative ectocommensal association reported here of *Heteromysis octopodis* with *Octopus vulgaris* represents the first documented regular association between mysids and cephalopods. On the first glance it sounds surprising that a commensal of sublittoral invertebrates may be also found in tide pools. However, there is a comparable case already known from *Heteromysis*: the E. Atlantic and Mediterranean *H. microps* G.O. Sars, 1877, is usually found on sublittoral mud flats, pebble bottoms, and in seagrass stands (Tattersall and Tattersall 1951, Zouhiri et al. 1998, Wittmann 2001), but also occurs on intertidal mud flats where it hovers in burrows of the mud shrimp *Upogebia pusilla* (Petagna, 1792) during low tide (Lavesque et al. 2016). The Y-shaped burrows penetrate up to 1 m into the sediment and serve as shelter to a diversity of invertebrate and fish species (listed by Lavesque et al. 2016). In analogy, there is several crustacean species associated with *Octopus* as is shown in Fig. 9B: in addition to *H. octopodis* there are an yet undetermined mysid species and the crab *Guinusia chabrus* (Linnaeus, 1758). So far, we did not examine whether these last crustaceans may regularly occur in such associations.

*H. cancelli* sp. n. follows *H. dardani* Wittmann, 2008, as the second association between mysids and hermit crabs documented from the E. Atlantic Ocean. The new species was found only in a large gastropod shell inhabited by *Cancellus macrothrix*, and not in any other type of microhabitat, although a variety of sampling methods was used. In order to test whether this commensalism may be obligate, a number of dead gastropod and sea urchin shells were collected 5.5 km from the type locality and then brought to the lab where the mysids were extracted and photographed still alive. Surprisingly, the shells yielded no *H. cancelli*, but only a different species described above as *H. fosteri* sp. n. (i.e. finding mysids in these empty shells was not surprising, but the exclusive occurrence of a different congeneric species in such shells was surprising). This points to obligate commensalism of the former species with hermit crabs, but insufficient data are available at present to demonstrate this, in that all specimens so far known derive from a single hermit specimen (among only five of this rare hermit crab species examined). The species has not, however, been observed amongst hundreds of hermit crabs of other species examined both at this site and in the broader region. With this new species a total of eight *Heteromysis* species have been reported as commensals of hermit crabs at a world-wide scale (Bonnier and Pérèz 1902, Clarke 1955, Tattersall 1967, Vannini et al. 1993, 1994, Fukuoka 2005, Wittmann 2008). As far as known, five *Heteromysis* species appear to be obligate commensals, at least two species are less host specific and are also found in microhabitats other than shells inhabited by hermit crabs. Based on morphological comparison Wittmann (2008) concluded that commensalism with hermit crabs may have independently evolved in the shelf areas of the Indo-Pacific, E. Pacific, and Atlantic oceans.

### Key to the *Heteromysis* species of the E. Atlantic

Modified and updated from the key given by Wittmann and Wirtz (2017). Certain heterogeneities in the structure of the key are due to missing data.

**Table:** 

	Heteromysini with normal eyes and a well-developed cornea. Males without mediocaudal lobe on appendix masculina of the antennular trunk; both sexes without a pair of backwards-oriented modified setae on inner terminal angle of antennular trunk, only forwards-oriented setae are present, not modified or modified in different ways. Gnathopod moderately to strongly stout, propodus separated from carpus by an oblique or a transverse articulation or not separated (propodus not always stouter than merus as in the three species described above). Thoracic endopod 4 with entire, apically not bifid claw. Thoracic endopod 8 in both sexes without flagellate spine on ischium and without spiniform extension of praeischium. Penes terminally not or only weakly widened by lobes. Females without sternal plate transversely projecting behind marsupium. Pleopods rudimentary and dimorphic or non-dimorphic. Genus *Heteromysis* S.I. Smith, 1873	**1**
1	Eyestalks without spiniform extension, or distally with small blunt extension of inner margin. All pleopods of male (if known) without spines or spine-setae	**5**
–	Eyestalks distally with small spiniform extension of the inner margin. At least one among third and fourth (usually both) pleopods of male with flagellate spines (spine-setae) distally on outer margin of endopodal portion	**2**
2	Apical cleft 22-23% telson length. Antennular trunk with two forwards-directed, smooth setae on inner, distal corner of terminal segment; no flagellate seta present. Endopod of uropods with 3-4 spines near statocyst. Only one damaged male known	***H. tattersalli* H. Nouvel, 1942** (Cape Verde Islands, 91 m)
–	Apical cleft longer than 23% telson length. One flagellate, strong, modified seta on inner distal corner of terminal segment of antennular trunk	**3**
3	Second male pleopod without spines. Endopod of uropods with 2–3 spines on inner margin near statocyst	**H. (Olivemysis) sabelliphila Wittmann & Wirtz, 2017** (Cape Verde Islands, 6–18 m, associated with sabellids, also on gravel bottom or in submarine cave)
–	Second male pleopod with one or more flagellate spines. Endopod of uropods with 3–4 spines	**4**
4	Fifth to seventh thoracic endopods with (4-6)-segmented carpopropodus. Endopod of uropods with three spines on inner margin near statocyst	**H. (Olivemysis) dardani Wittmann, 2008** (Madeira, 10–20 m, commensal of hermit crab)
–	Fifth to seventh thoracic endopods with seven-segmented carpopropodus. Endopod of uropods with four spines on inner margin near statocyst	**H. (Olivemysis) wirtzi Wittmann, 2008** (Madeira, 28 m, commensal of sea anemone)
5	Telson with spines along distal 80-100% of the lateral margins	**10**
–	Telson with spines mainly on distal half; without spines, or at most one spine, on basal third of each lateral margin	**6**
6	Telson cleft wider than deep. Endopod of uropods with four spines on inner margin near statocyst. Powerful gnathopod with stout carpus. Only one female specimen known	***H. atlantidea* O.S. Tattersall, 1961** (Cape Verde Islands, 8 m)
–	Telson cleft at least about as deep as wide, in most species deeper than wide	**7**
7	Telson with one spine on basal third of each lateral margin, followed by a naked portion and then 8-13 spines on distal half; medio-apical spines distinctly shorter than latero-apical spines. Endopod of uropods with only one spine, which is near statocyst	**H. (Heteromysis) microps (G.O. Sars, 1877)** (NE Atlantic and Mediterranean: N Adriatic, Gulf of Naples, Sardinia, Tunisia, 0–16 m, mud flats, gravel bottoms, seagrass stands, burrows of mud shrimp, meso- to metahaline)
–	Telson without spines on basal third of lateral margins	**8**
8	Telson on each side with one medio-apical spine being distinctly shorter than latero-apical spine; cleft with a total of 26–37 laminae all along its margins. Endopod of uropods with only one spine below statocyst	**H. (Heteromysis) octopodis sp. n.** (South Africa: Cape Peninsula, 0-3 m, associated with octopus or in tide pools)
–	Telson with the 1–2 medio-apical spines on each side longer or subequal compared to latero-apical spines	**9**
9	Endopod of uropods with only one spine below statocyst. Telson cleft densely furnished with a total of 29–33 laminae along basal 81–84% of its margins	**H. (Heteromysis) cancelli sp. n.** (South Africa: Cape Peninsula, 20 m, associated with hermit crab)
–	Endopod of uropods with 17-30 spines along inner margin between statocyst and tip. Telson cleft densely furnished with a total of 11–21 laminae along basal 50–70% of its margins	**H. (Heteromysis) norvegica G.O. Sars, 1882** (NE Atlantic and Mediterranean, west coast of Norway, British Isles, English Channel, north and west coasts of France, Portugal, Madeira, Atlantic coast of Morocco, Mediterranean: off Monaco, Malta, 0–400 m)
10	Endopod of uropods with only one spine below statocyst. Telson cleft with 16-24 laminae all along its margins	**H. (Heteromysis) fosteri sp. n.** (South Africa: Cape Peninsula, 4 m, in empty shells)
–	Endopod of uropods with 20–24 spines in dense series along inner margin. Telson cleft with 10-12 laminae only on its proximal half	**H. (Heteromysis) armoricana H. Nouvel, 1940** (coast of Brittany, Galicia, Mediterranean, shallow coastal waters, muddy sand bottom, brown algae)

## Supplementary Material

XML Treatment for Heteromysis (Heteromysis) cancelli

XML Treatment for Heteromysis (Heteromysis) fosteri

XML Treatment for Heteromysis (Heteromysis) octopodis

## References

[B1] Băcescu M (1986) Two new species of *Heteromysis* from the coral reefs of northern Australia. Travaux du Muséum d´Histoire Naturelle "Grigore Antipa" 28: 19–24. https://www.travaux.ro/web/pdf/28-TMNHNGA-19-24.pdf [accessed on 2015-12-19]

[B2] Bergh RS (1893) Beiträge zur Embryologie der Crustaceen. 1. Zur Bildungsgeschichte des Keimstreifens von *Mysis*. Zoologische Jahrbücher, Abtheilung für Anatomie und Ontogenie der Thiere 6: 491–528, pls 26–29.

[B3] Bonnier J Pérez C (1902) Sur un Crustacé commensal des Pagures, *Gnathomysis Gerlachei*, nov. sp., type d’une famille nouvelle des Schizopodes. Comptes Rendus Hebdomadaires des Séances de l´Académie des Sciences 134: 117–119.

[B4] Bousfield EL (1958) Littoral marine arthropods and mollusks collected in Western Nova Scotia, 1956. Proceedings of the Nova Scotian Institute of Science 24(3): 303–325. http://hdl.handle.net/10222/13549 [accessed on 2017-05-14]

[B5] Carreño I Pujol S Zoppi de Roa E (1989) *Metamysidopsis insularis* Brattegard (Mysidacea): Field study of the larval cycle. Crustaceana 56(2): 127–131. https://doi.org/10.1163/156854089X00022

[B6] Clarke WD (1955) A new species of the genus *Heteromysis* (Crustacea, Mysidacea) from the Bahama Islands, commensal with a sea-anemone. American Museum Novitates 1716: 1–13. http://hdl.handle.net/2246/4712 [accessed on 2017-05-14]

[B7] Connell AD (1974) Mysidacea of the Mtentu River Estuary, Transkei, South Africa. Zoologica Africana 9(2): 147–159. https://doi.org/10.1080/00445096.1974.11448522

[B8] Davis CC (1966) A study of the hatching process in aquatic invertebrates. XXII. Multiple membrane shedding in *Mysidium columbiae* (Zimmer) (Crustacea: Mysidacea). Bulletin of marine Science 16(1): 124–131. http://www.ingentaconnect.com/contentone/umrsmas/bullmar/1966/00000016/00000001/art00007 [accessed on 2017-05-14]

[B9] Frutos I Sorbe JC (2013) Bathyal suprabenthic assemblages from the southern margin of the Capbreton Canyon (“Kostarrenkala” area), SE Bay of Biscay. Deep-Sea Research Part II: Topical Studies in Oceanography 102: 291–309. https://doi.org/10.1016/j.dsr2.2013.09.010i

[B10] Fukuoka K (2005) A new species of *Heteromysis* (Mysida, Mysidae) associated with sponges, from the Uraga Channel, central Japan, with notes on distribution and habitat within the genus *Heteromysis*. Crustaceana 77(11): 1353–1373. https://doi.org/10.1163/1568540043165976

[B11] Green JM (1970) Observations on the behavior and larval development of *Acanthomysis sculpta* (Tattersall, Mysidacea). Canadian Journal of Zoology 48: 289–292. https://doi.org/10.1139/z70-047 5415447 10.1139/z70-047

[B12] Jepsen J (1965) Marsupial development of *Boreomysis arctica* (Krøyer, 1861). Sarsia 20: 1–8. https://doi.org/10.1080/00364827.1965.10409550

[B13] Jirikowski GJ Wolff C Richter S (2015) Evolution of eumalacostracan development — new insights into loss and reacquisition of larval stages revealed by heterochrony analysis. EvoDevo 6(4): 1–30. https://doi.org/10.1186/2041-9139-6-4 25973168 10.1186/2041-9139-6-4 PMC4429915

[B14] Klepal W Kastner RT (1980) Morphology and differentiation of non-sensory cuticular structures in Mysidacea, Cumacea and Tanaidacea (Crustacea, Peracarida). Zoologica Scripta 9: 271–281. https://doi.org/10.1111/j.1463-6409.1980.tb00667.x

[B15] Lagardère JP Nouvel H (1980) Les mysidacés du talus continental du golfe de Gascogne. II. Familles des Lophogastridae, Eucopiidae et Mysidae (Tribu des Erythropini exceptée). (Suite et fin). Bulletin du Muséum national d’Histoire naturelle, 4ème série, section A (Zoologie, Biologie, Écologie animale) 2(3): 845–887. http://www.vliz.be/imisdocs/publications/282458.pdf [accessed on 2017-05-05]

[B16] Lavesque N Pascal L Gouilleux G Sorbe J-C Bachelet G Maire O (2016) Heteromysis (Heteromysis) microps (Crustacea, Mysidae), a commensal species for *Upogebia pusilla* (Crustacea, Upogebiidae) in Arcachon Bay (NE Atlantic Ocean). Marine Biodiversity Records 9(14): 1–6. https://doi.org/10.1186/s41200-016-0001-1

[B17] Manton SM (1928) On the embryology of a mysid crustacean, *Hemimysis lamornae*. Philosophical Transactions of the Royal Society. B. Biological Sciences 216: 363–463. [pls 21–25] https://doi.org/10.1098/rstb.1928.0008 10.1098/rstb.2014.0381 PMC4360129 25750244

[B18] Mees J [Ed.] (2017) Mysida World Register of Marine Species. http://www.marinespecies.org/aphia.php?p=taxlist [accessed on 2017-05-28]

[B19] Modlin RF (1979) Development of *Mysis stenolepis* (Crustacea: Mysidacea). The American Midland Naturalist 101(1): 250–254. https://doi.org/10.2307/2424923

[B20] Nusbaum J (1887) L’embryologie de *Mysis chameleo* (Thompson). Archives de Zoologie expérimentale et générale, ser. 2: 5: 123–202. [pls V-XII] http://gallica.bnf.fr/ark:/12148/bpt6k5455965b.image.langEN [accessed on 2012-01-08]

[B21] Price WW Heard RW (2011) Two new species of Heteromysis (Olivemysis) (Mysida, Mysidae, Heteromysinae) from the tropical northwest Atlantic with diagnostics on the subgenus Olivemysis Băcescu, 1968. Zootaxa 2823: 32–46. https://doi.org/10.5281/zenodo.207036

[B22] San Vicente C Monniot F (2014) The ascidian-associated mysid *Corellamysis eltanina* gen.nov., sp.nov. (Mysida, Mysidae, Heteromysinae): a new symbiotic relationship from the Southern Ocean. Zootaxa 3780(2): 323–346. https://doi.org/10.11646/zootaxa.3780.2.6 24871839 10.11646/zootaxa.3780.2.6

[B23] Takahashi K Kawaguchi K (1997) Diel and tidal migrations of the sand-burrowing mysids, *Archaeomysis kokuboi*, *A. japonica* and *Iiella ohshimai*, in Otsuchi Bay, northeastern Japan. Marine Ecology Progress Series 148: 95–107. http://www.jstor.org/stable/24857475 [accessed on 2017-05-05]

[B24] Tattersall OS (1967) A survey of the genus *Heteromysis* (Crustacea: Mysidacea) with descriptions of five new species from tropical coastal waters of the Pacific and Indian Ocean, with a key for the identification of the known species of the genus. Transactions of the Zoological Society of London 31: 157–193. https://doi.org/10.1111/j.1096-3642.1967.tb00366.x

[B25] Tattersall WM Tattersall OS (1951) The British Mysidacea. Ray Society, London, monograph no. 136, 460 pp http://www.pisces-conservation.com/softmys.html [accessed on 2010-04-27]

[B26] Vannini M Innocenti G Ruwa RK (1993) Family group structure in mysids, commensals of hermit crabs (Crustacea). Tropical Zoology 6: 189–205. https://doi.org/10.1080/03946975.1993.10539219

[B27] Vannini M Ruwa RK Innocenti G (1994) Notes on the behaviour of *Heteromysis harpax*, a commensal mysid living in hermit crab shells. Ethology Ecology & Evolution (Spec. Issue) 3: 137–142. https://doi.org/10.1080/03949370.1994.10721987

[B28] Wittmann KJ (1981) Comparative biology and morphology of marsupial development in *Leptomysis* and other Mediterranean Mysidacea (Crustacea). Journal of experimental marine Biology and Ecology 52(2-3): 243–270. https://doi.org/10.1016/0022-0981(81)90040-X

[B29] Wittmann KJ (2000) *Heteromysis arianii* sp.n., a new benthic mysid (Crustacea, Mysidacea) from coralloid habitats in the Gulf of Naples (Mediterranean Sea). Annalen des Naturhistorischen Museums in Wien 102 (B): 279–290. http://www.zobodat.at/pdf/ANNA_102B_0279-0290.pdf [accessed on 2013-07-15]

[B30] Wittmann KJ (2001) Centennial changes in the near-shore mysid fauna of the Gulf of Naples (Mediterranean Sea), with description of *Heteromysis riedli* sp. n. (Crustacea, Mysidacea). Marine Ecology 22(1–2): 85–109. https://doi.org/10.1046/j.1439-0485.2001.00741.x

[B31] Wittmann KJ (2008) Two new species of Heteromysini (Mysida, Mysidae) from the Island of Madeira (N.E. Atlantic), with notes on sea anemone and hermit crab commensalisms in the genus *Heteromysis* S. I. Smith, 1873. Crustaceana 81(3): 351–374. https://doi.org/10.1163/156854008783564037

[B32] Wittmann KJ Ariani AP Lagardère JP (2014) Orders Lophogastrida Boas, 1883, Stygiomysida Tchindonova, 1981, and Mysida Boas, 1883 (also known collectively as Mysidacea). In: von Vaupel Klein JC Charmantier-Daures M Schram FR (Eds) Treatise on Zoology - Anatomy, Taxonomy, Biology. The Crustacea. Revised and updated, as well as extended from the Traité de Zoologie 4 Part B (54), 189–396. [colour plates: 404–406] https://doi.org/10.1163/9789004264939_006

[B33] Wittmann KJ Griffiths CL (2014) Description of the ‘stargazer mysid’ *Mysidopsis zsilaveczi* sp. nov. (Mysida: Mysidae: Leptomysinae) from the Cape Peninsula, South Africa. Crustaceana 87(11): 1411–1429. https://doi.org/10.1163/15685403-00003364

[B34] Wittmann KJ Schlacher TA Ariani AP (1993) Structure of Recent and fossil mysid statoliths (Crustacea, Mysidacea). Journal of Morphology 215: 31–49. https://doi.org/10.1002/jmor.1052150103 10.1002/jmor.1052150103 29865427

[B35] Wittmann KJ Wirtz P (2017) *Heteromysis sabelliphila* sp. nov. (Mysida: Mysidae: Heteromysinae) in facultative association with sabellids from the Cape Verde Islands (subtropical N.E. Atlantic). Crustaceana 90(2): 131–151. https://doi.org/10.1163/15685403-00003542

[B36] Zouhiri S Vallet C Mouni P Dauvin J-C (1998) Spatial distribution and biological rhythms of suprabenthic mysids from the English Channel. Journal of the Marine Biological Association of the United Kingdom 78: 1181–1202. https://doi.org/10.1017/S0025315400044416

